# CXCL8 is essential for cervical cancer cell acquired radioresistance and acts as a promising therapeutic target in cervical cancer

**DOI:** 10.1038/s41598-025-05435-w

**Published:** 2025-07-01

**Authors:** Qinghong Hu, Xiaoxiao Zuo, Xiaobin Gu, Liya Liu, Ying Tang, Xiaomin Niu, Yonggang Shi, Liping Han

**Affiliations:** 1 Department of Radiation Oncology, The First Affiliated Hospital of Zhengzhou University, Zhengzhou, 450000 China; 2 Department of Gynecology, The First Affiliated Hospital of Zhengzhou University, Zhengzhou, 450000 China

**Keywords:** Cervical cancer, Radioresistance, CXCL8, Radiosensitization, Tumor microenvironment, Cancer, Biomarkers, Cancer models, Gynaecological cancer, Tumour biomarkers, Cancer therapy, Cancer therapeutic resistance, Drug development, Radiotherapy, Oncology

## Abstract

Acquired radioresistance critically challenges cervical cancer radiotherapy management. Clinically relevant radioresistant cell models remain scarce, and CXCL8’s role in cervical cancer—despite its tumorigenic/therapy-resistant associations in other cancers—is poorly characterized. Two radioresistant cervical cancer cell strains were established. mRNA-seq and bioinformatics analysis of radiosensitivity regulators identified CXCL8 as a key mediator. In vitro, assays of cell viability, clone formation, apoptosis and cell cycle were conducted following transient transfection of cervical cancer radiotherapy-resistant cell strains with knockdown of CXCL8, as well as subsequent addition of exogenous CXCL8 to cervical cancer parental cell strains. Radioresistant cervical cancer cell lines (Hela-RR/Siha-RR) were established through clinical protocol-mimicking irradiation, validated via proliferation/clonogenic/cell cycle assays. mRNA-seq identified 50 co-upregulated and 54 co-downregulated genes in resistant strains, with CXCL8 among top differentially expressed genes (IL11, CXCL8, MMP1, HSPA8, CA9, PPFIA4, EDN2, GUCY1A2, EFNA3, TNFAIP6). qRT-PCR confirmed CXCL8, TNFAIP6, SRNA8 and PPFIA4 dysregulation. Cox regression analysis of 96 candidate radiosensitivity regulators prioritized CXCL8 among eight key genes in cervical cancer. GEPIA2 and immunohistochemistry revealed CXCL8 overexpression in tumors. Functional studies demonstrated CXCL8 knockdown sensitized resistant cells to radiation, while exogenous CXCL8 induced resistance in parental lines.

**Supplementary Information:**

The online version contains supplementary material available at 10.1038/s41598-025-05435-w.

## Introduction

Cervical cancer (CC), the most prevalent gynecological malignancy, remains a major global health issue, contributing significantly to disability-adjusted life years^1^. In 2020, an estimated 604,127 new cases and 341,831 deaths from CC were reported worldwide, making it the fourth most common cancer among women^2^. While incidence rates have decreased in Europe and the United States, they remain high in Hispanic, Latino, and Asian populations^3^. Prevention relies heavily on screening and immunization, with 88.5% of cases occurring in low-income countries, where CC remains the leading cause of cancer-related deaths among women^4^. Due to its often asymptomatic nature and poor healthcare access, over two-thirds of patients present with locally advanced or metastatic disease at diagnosis. The standard treatment for locally advanced CC (LACC) involves cisplatin-based concurrent chemoradiotherapy, with a 5-year survival rate ranging from 20 to 65%, a result widely considered suboptimal^5,6^. CC continues to pose a significant threat to women’s health worldwide^7^.

Radiotherapy plays a critical role in treating CC by using X-rays and β-rays to eliminate tumor cells while preserving organ function. The National Comprehensive Cancer Network (NCCN) recommends external beam radiotherapy combined with brachytherapy and platinum-based chemotherapy for patients with stage IB3, II, III, and IVA CC^8^. Although this multimodal approach improves progression-free survival and overall survival, outcomes frequently fall short of expectations due to uncontrolled tumor growth, recurrence, and distant metastasis^9^.

Radiotherapy resistance represents the principal obstacle to effective CC treatment^10^. This resistance can be classified into innate and acquired forms, referring to the tumor cells’ relative resistance to the biological effects of radiotherapy at cellular, tissue, organ, or organismal levels^11^. Although the mechanisms underlying radiotherapy resistance in malignancies remain largely unclear, potential factors include tumor hypoxia^12^, tumor microenvironment (TME)^13^, DNA damage repair^14^, cancer stem cells^15^, metabolic reprogramming^16^, cell cycle regulation, apoptosis, and other signaling pathways^17^. In CC, the mechanisms of radiotherapy resistance are particularly complex, with TME alterations playing a critical role. Dysfunctional TMEs promote anti-apoptotic pathways and enhance resistance to radiotherapy through various mechanisms. Studies suggest that CC cells’ resistance to radiotherapy may be driven by specific signals, such as antigens and cytokines, within the TME^18,19^. Therefore, the primary challenge in CC radiotherapy is the development of acquired resistance. Overcoming or mitigating this resistance through targeted strategies is a key research focus, aiming to improve the efficacy of CC treatments.

C-X-C motif chemokine ligand 8 (CXCL8), also known as interleukin-8^20^, was initially identified as a pro-inflammatory chemokine. However, within the TME, it has been found to be produced by various cell types, including infiltrating immune cells, stromal cells, and tumor cells^21^. Recent studies have shown that CXCL8 expression is significantly upregulated in several cancers, including prostate cancer, colorectal cancer, head and neck squamous cell carcinoma, osteosarcoma, and glioma. This chemokine is strongly associated with processes such as tumor proliferation, invasion, migration, angiogenesis, cancer stem cells (CSCs), and immune evasion^22,23^. Emerging evidence indicates that CXCL8 plays a critical regulatory role in the TME, contributing to tumor progression and metastasis, and can directly promote resistance to chemotherapy, targeted therapies, and immune checkpoint inhibitors (ICIs)^22–25^. While CXCL8’s involvement in cancer progression and drug resistance has been extensively studied, its role in radiotherapy resistance in CC remains unexplored.

Two distinct acquired radiotherapy-resistant CC cell lines were developed through repeated intermittent irradiation, mimicking clinical treatment patterns. High-throughput mRNA sequencing was employed to profile differential mRNA expression, and bioinformatics analysis was conducted to investigate the underlying molecular mechanisms. Additionally, bioinformatics tools identified key genes involved in regulating radiosensitivity and resistance to radiation therapy in CC. Potential molecular targets for overcoming radiation resistance in CC were subsequently screened. Based on these findings, CXCL8 was identified as a critical regulator of acquired radiotherapy resistance. In vitro experiments were carried out to explore the role and molecular mechanism of CXCL8 in radiotherapy resistance. It is expected that further elucidation of the molecular mechanisms governing resistance will facilitate the identification of effective sensitization targets and inform strategies for reversing resistance in CC.

Acquired radioresistance critically impedes cervical cancer therapy. While CXCL8, a pro-tumor chemokine, is implicated in cancer progression and treatment resistance across malignancies, its role in cervical cancer radioresistance remains uncharacterized. Targeting its molecular mechanisms may provide novel radiosensitizing strategies to overcome therapeutic resistance.

## Materials and methods

### Cell culture and irradiation

All experiments, including any relevant details, were approved by the Ethics Committee of The First Affiliated Hospital of Zhengzhou University (Approval No. 2023-KY-0570-002).All methods were performed in accordance with relevant guidelines and regulations. The human cervical cancer cell lines Hela and Siha, were obtained from American Type Culture Collection (ATCC, USA). All the cell lines were maintained in Dulbecco’s Modified Eagle Medium (HyClone, USA) containing 10% fetal bovine serum (Gibco, USA) and 1% penicillin-streptomycin at 37 °C and 5% CO2. The cells were irradiated with 2 Gy of 6 MV-X rays using a high linear accelerator (Varian, USA) at a dose rate of 300 cGy/min, a field size of 20 cm×20 cm, a source skin distance adjustment of 100 cm, with tissue compensation membrane. Following irradiation, cells were returned to the incubator. Irradiation (2 Gy) was repeated every five days, after which the cells were cultured to facilitate recovery. Following this, the cells were passaged and irradiated once more upon reaching 75% fusion and the portion of the cells was preserved during the irradiation process and labelled with the respective time and cumulative dose. It took nearly a year to duplicate the cervical cancer cell lines, named Hela-RR (Hela radioresistance) and Siha-RR (Siha radioresistance), which are genetically stable and tolerant to 6 MV-X rays at 60 Gy. Hela-RR and Siha-RR were cultured under the same conditions as Hela and Siha, and the selected irradiation pressure was 2 Gy/week. Hela and Siha cells were selected for irradiation with 6 MV-X-rays at doses of 2, 4, 6, 8 and 10 Gy, respectively, once the logarithmic growth time was reached and the fusion ratio reached 75%. Cells capable of five consecutive generations were selected for retention and further passage. During the irradiation process, it was observed that Hela cells were unable to stably pass on under conditions of irradiation at doses of 8 Gy or higher. This finding indicated that a dose of 6 Gy was identified as the single sublethal dose for Hela cells. The same experimental method was employed in subsequent studies. This method was also employed in the treatment of Siha cells, and the results indicated that Siha cells were unable to stably survive at doses of 6 Gy or higher. This finding suggests that 4 Gy was the single sublethal dose for Siha cells.

### Colony formation assay

Logarithmically grown cells (Hela, Hela-RR cells, Siha and RSiha-RR) were grouped according to the dose gradient 0 Gy, 2 Gy, 4 Gy, 6 Gy, 8 Gy, 10 Gy, and different cells were inoculated in the six-well plates. 0 Gy/1000, 2 Gy/2000, 4 Gy/4000, 6 Gy/6000, 8 Gy/8000, and 10 Gy/10,000. The experiment was replicated thrice. Following cell attachment to the well wall of the six-well plate, 6MV-X-ray irradiation was performed, with the dose set according to the dose gradient. The irradiation formula is as follows: As previously mentioned, the culture was placed in the incubator with a CO2 concentration of 5% at 37 °C. After 14 days, the number of colonies was counted after staining and drying by 4% paraformaldehyde and crystal violet dye. The cells within each colony must reach or exceed 50. The survival fraction curve was drawn based on the multi-target click model [SF = 1-(1-e-D/D0) N], and the curve was drawn and fitted using Graphpad 9.0 software.

### Time-dependent cell proliferation assay after irradiation

Logarithmically grown cells (Hela, Hela-RR cells, Siha and Siha-RR) were digested and counted and diluted to contain 3,000 cells per 100 µl and then seeded in 96-well plates with 4,000 cells per well and 3 replicate wells, and the blank control group was set up with medium that did not contain cells. The cells were divided into unirrdiated group and irradiated group according to whether they received radiation irradiation or not, and the irradiated group was irradiated with 6 Gy for Hela and Hela-RR cells and 4 Gy for Siha and Siha-RR cells, and the culture was continued by changing the fluid after irradiation. The irradiated group was assessed on days 1, 2, 3, 4, 5, 6, and 7 after irradiation; the unirradiated group was tested on days 1, 2, 3, 4, and 5 after plate laying; 10 µl of CCK-8 reagent was added to each group of cells as well as the blank control wells in the incubator at the same time every day for 1 h, and then an enzyme countermeasure was used to measure the OD value at the position of 450 nm. In the unirradiated group, the OD value of the cells on the first day was regarded as A0; the OD value of the irradiated group that was not affected by radiation before irradiation was regarded as A0, and the cell survival rate at different time points was calculated: cell survival rate = (An-A blank)/ (A control well-A blank) × 100%.

### Apoptosis assay by flow cytometry

Logarithmically grown Hela and Hela-RR cells were irradiated with 6 Gy, Siha and Siha-RR cells with 4 Gy. After irradiation, the cells continued to be cultured after changing the medium at 5% CO2 concentration and 37 °C. After 24 h, the cells were washed twice with cold PBS, resuspended in 1X Binding Buffer (1 × 10^6^ cells/ml), and aliquoted (100 µl; 1 × 10⁵ cells) into 5 ml culture tubes. They were stained with 5 µl FITC Annexin V and 5 µl PI (BD, USA), gently vortexed, incubated for 15 min at 25 °C in the dark, diluted with 400 µl 1X Binding Buffer, and analyzed by CytoFLEX flow analysis (Beckman Coulter, USA) within 1 h. The following controls are used to set up compensation and quadrants: unstained cells, cells stained with FITC Annexin V (no PI) and cells stained with PI (no FITC Annexin V).

### Cell cycle assays by flow cytometry

Cells were selected for logarithmic growth and inoculated in 6-well plates at a density of 4 × 10^5^/well. Hela and Hela-RR cells were treated with 6 Gy radiation or without, and Siha and Siha-RR cells were treated with 4 Gy radiation or without. After irradiation, the cells continued to be cultured after changing the medium at 5% CO_2_ concentration and 37 °C. Hela and Hela-RR cells, Siha and Siha-RR cells (unirradiated, 1 h after irradiation, and 12 h after irradiation) and the supernatants were collected for subsequent processing. The cells and the supernatants were pelleted and washed with PBS. Cells were fixed in 75% ethanol and stained with propidium iodide (KeyGEN, China), followed by by the CytoFLEX flow analysis (Beckman Coulter, USA).

### Cell invasiveness assay

Matrigel (BD, USA) dilution was meticulously applied to the upper chamber of the membrane, which was positioned at the base of the chambers. The suspension was then prepared: cells were subjected to a 12-hour serum starvation period, followed by a resuspension in serum-free medium, yielding a density of 5 × 105 cells/ml. / ml. Then, 100 µl of the cell suspension was added to the chambers. Next, 600 µl of medium was added to the lower chamber, and the cells were cultured for 12 h. Then, the cells were fixed in methanol and stained with crystalline violet. The counts were observed under a 400x microscope by switching to five different viewing angles at random.

### mRNA-seq technology

Total RNA was isolated using TRIzol reagent (Thermo Fisher Scientific, USA), after which the RNA concentration and quality were determined by the Qubit^®^3.0 Fluorometer (Life Technologies, USA) and the Nanodrop One spectrophotometer (Thermo Fisher Scientific Inc., USA). Integrity of total RNA was assessed using the Agilent 2100 Bioanalyzer (Agilent Technologies Inc., USA), and samples with RNA integrity number (RIN) values above 7.0 were used for sequencing Hela-RR and Hela, Siha-RR and Siha. One micrograms of RNA were used as input material for the RNA sample preparations. Paired-end libraries were synthesized by using the Stranded mRNA-seq Lib Prep Kit for Illumina (ABclonal, China) following the Preparation Guide. Briefly, the poly-A containing mRNA molecules were purified using poly-T oligo-attached magnetic beads. Following purification, the mRNA is fragmented into small pieces using divalent cations under 94 ℃ for 10 min. The cleaved RNA fragments are copied into first strand cDNA using reverse transcriptase and random primers. This is followed by second strand cDNA synthesis using DNA Polymerase I and RNase H. These cDNAfragments then go through an end repair process, the addition of a single ‘A’ base, and then ligation of the adapters. The products are then purified and enriched with PCR to create the final cDNA library. Purified libraries were quantified by Qubit^®^ 3.0 Fluorometer (Life Technologies, USA) and validated by Agilent 2100 bioanalyzer (Agilent Technologies, USA) to confirm the insert size and calculate the mole concentration. Cluster was generated by cBot with the library diluted to 10 pM and then were sequenced on the Illumina NovaSeq 6000 (Illumina, USA). The library construction and sequencing were performed by Sinotech Genomics Co. Ltd (Shanghai, China). We used R (v4.2.1) for differential expression analysis, pathway enrichment, and heatmap visualization.

### Real-time PCR

Total RNA was isolated utilizing the TRIzol reagent (Thermo Fisher Scientific, USA). From the extracted total RNA, cDNA synthesis was performed with the Evo M-MLV RT Master Mix (Agencourt Biology, China). qRT-PCR was then performed utilizing ChamQ Universal SYBR qPCR Master Mix (Vazyme, China) on ABI 7500 FAST Real-Time PCR system (Applied Biosystems, Waltham, MA, UK). GAPDH served as the housekeeping gene. Calculation of the relative expression level was performed according to 2 − ΔΔCt formula, and synthesis of primers was supported by GenePharma (Shanghai China). The real-time PCR primer sequences are given in Supplementary Table 1.

### Training set queue data acquisition and processing

Clinical information and expression data for CESC were obtained from the TGCA-GTEx-CESC dataset on the website https://xenabrowser.net/datapages/ Differential analyses were performed using TCGA samples for cancer samples and GTEx data samples for control samples, and except for the differential analyses, the training set was only analysed in the subsequent analysis non-duplicated TCGA samples containing survival information were used. For subsequent studies, we retained female tumour samples containing both expression and survival information for in-depth analysis (291 samples).

### Validation queue data acquisition and processing

Scoring system was validated using the GPL6244 platform GSE52903 dataset based on the GEO database (https://www.ncbi.nlm.nih.gov/geo/). Expression profiling data and clinical data were downloaded from the GEO database: https://www.ncbi.nlm.nih.gov/geo. If duplicate genes were encountered, the median expression value was taken as the final expression. During GEO data processing: (1) pairs of null-loaded probes were removed. (2) When a probe corresponds to more than one gene, the probe was deleted. (3) When multiple probes are able to correspond to the detection of the same gene, we will use the median of the values expressed by these probes, which will be used as the expression value for this gene.

### Cancer radiosensitivity regulation factors database

A total of 395 genes related to Cancer Radiosensitivity Regulation Factors (CRRF) were obtained from the dbCRSR database (Cancer Radiosensitivity Regulation Factors Database).

### Score construction

In the training set cohort, 291 primary cancer patient samples were screened for differential genes between cancer and paraneoplastic using the R package limma, and then intersections were taken with radiosensitivity modifiers to obtain radiosensitivity modifiers that were differentially expressed in CESC. The differentially expressed radiosensitivity regulators were subjected to one-way cox regression with the threshold set at *p <* 0.05 to obtain significant prognostic factors. Using the significant prognostic factors, lasso-cox analysis was performed by the R package glmnet to calculate the correlation coefficients of each gene, and the patient scoring system was constructed according to Eq:

$$\:\text{S}\text{c}\text{o}\text{r}\text{e}=\sum\limits_{i=0}^{n}{\upbeta\:}\text{i}\text{*}{\upchi\:}\text{i}$$

βi: weight coefficient corresponding to each gene; χi: expression of each gene.

Based on the median score, we classified 291 primary cancer patients into two groups, the high-scoring group and the low-scoring group, and analysed the different prognoses of the high-scoring and low-scoring groups using the R package survminer for survival.

### CXCL8 expression and prognosis in GEPIA2

Expression levels of CXCL8 in cervical cancer tissues and paracancerous tissues were analysed by using a large dataset of GEPIA2 (Gene Expression Profiling Interactive Analysis, GEPIA) (gepia2.cancer-pku.co.uk/) in combination with TCGA and GTEx; and analysing CXCL8 in the survival analysis of high and low expression and prognosis of cervical cancer.

### Immunohistochemistry (IHC)

Paraffin-embedded cervical cancer tissue and non-tumoral cervical tissue specimens who underwent surgical treatment in our hospital from May 2023 to October 2023 (*n* = 12 for each group) were fixed in 4% paraformaldehyde. Informed consent was obtained from all participants and/or their legal guardians prior to tissue sample collection. IHC was conducted using anti-CXCL8 primary antibody (Bioss, China). Briefly, sections were deparaffinized, blocked using 0.3% hydrogen peroxide and goat serum sequentially, rinsed with a tris-buffered saline solution and incubated with the diluted primary antibody at 4 ℃ overnight. Following this, sections were gradually incu-bated with an HRP-labeled secondary antibody, stained with DAB (Agilent Technologies, USA), and applied hematoxy-lin for nuclear counterstaining. After dehydrating and mounting the sections, images were taken under an optical microscope. Using Image Pro Plus 5.0 software (Leica, Germany), the positive and total cell counts were deter-mined in five randomly chosen 400× microscopic fields.

Immunohistochemical scoring: the number of positive cells was counted by two randomly selected senior specialists in the Department of Pathology from each of the five different fields of view under a 400x microscope. Positive cells appeared as small yellow or brown granules. For each specimen, the total score of expression intensity (negative staining: 0; weak staining: 1; moderate staining: 2; and strong staining: 3) was multiplied by the stained cell number (positive cells as ≤ 25% of the cells, 1; 26–50% of the cells, 2; 51–75% of the cells, 3; > 75% of the cells, 4). When the sample was scored ≥ 7, we defined it as high expression, otherwise low expression.

### SiRNA transfection

Negative control and CXCL8-targeting siRNAs were synthesized by GenePharma (China) and transfected into Hela-RR and Siha-RR cell lines using Lipofectamine 3000 transfection reagent (Thermo Fisher Scientific, USA) and Opti-MEM serum-reduced medium (Thermo Fisher Scientific, USA) following the manufacturer’s instructions. Briefly, cells were transfected with 2 µg siRNA dissolved in DEPC water. 48 h later, the target RNA levels after transfection were verified by qRT-PCR. Cell viability assay, cell clone formation assay, cell apoptosis assay, and cell cycle assay were performed 48 h after transfection or 48 h after CXCL8 (final concentration 100 ng/mL) addition, 24 h after X-ray irradiation given at 6 Gy (Hela and Hela-RR) or 4 Gy dose (Siha and Siha-RR), followed by the cell viability assay, cell clone formation assay, apoptosis assay, and cell cycle assay. Sequences of these siRNAs are presented in Supplementary Table 2.

### Statistical analysis

GraphPad Prism version 9.0 and R (4.2.1) were used for statistics and visualization. Two-tailed Student’s t test, 1-way ANOVA, Pearson correlation analysis, Kaplan-Meier analysis, log-rank test and Cox’s proportional hazards regression model were applied to analyze the corresponding data. The combination score was determined using SynergyFinder. Statistical significance was assigned as ***P < = 0.001; **P < = 0.01; *P < = 0.05; NS (not sig-nificant) *P* > 0.05.

## Results

### Successful construction of two radiotherapy-resistant cell lines for cervical cancer

The study was conducted following the clinical treatment model, aiming to delineate the target area, plan the irradiation design, and verify the radiation dose delivered to the cells. Each irradiation cycle was completed with the application of Bolus, a tissue compensator, to ensure the precise delivery of the intended dose. The irradiation process is presented in Supplementary Fig. 1. Upon receiving a single 2 Gy dose for approximately five cycles, the CC HeLa cell line began to show gradual increases in cell size and morphological irregularity under microscopic observation. Similarly, the SiHa cell line, after receiving a single 2 Gy dose for four cycles, exhibited similar cellular expansion and morphological alterations over 7–9 days. The number of cell deaths peaked within this period, but surviving cells began to recover, with cell size, morphology, and proliferative capacity returning to normal and gradually enhancing over time. No significant morphological differences were observed between HeLa-RR and SiHa-RR cells under the microscope (Supplementary Fig. 2).

### Acquired radiotherapy-resistant cell lines have enhanced clone formation after radiation exposure

**Fig. 1:**
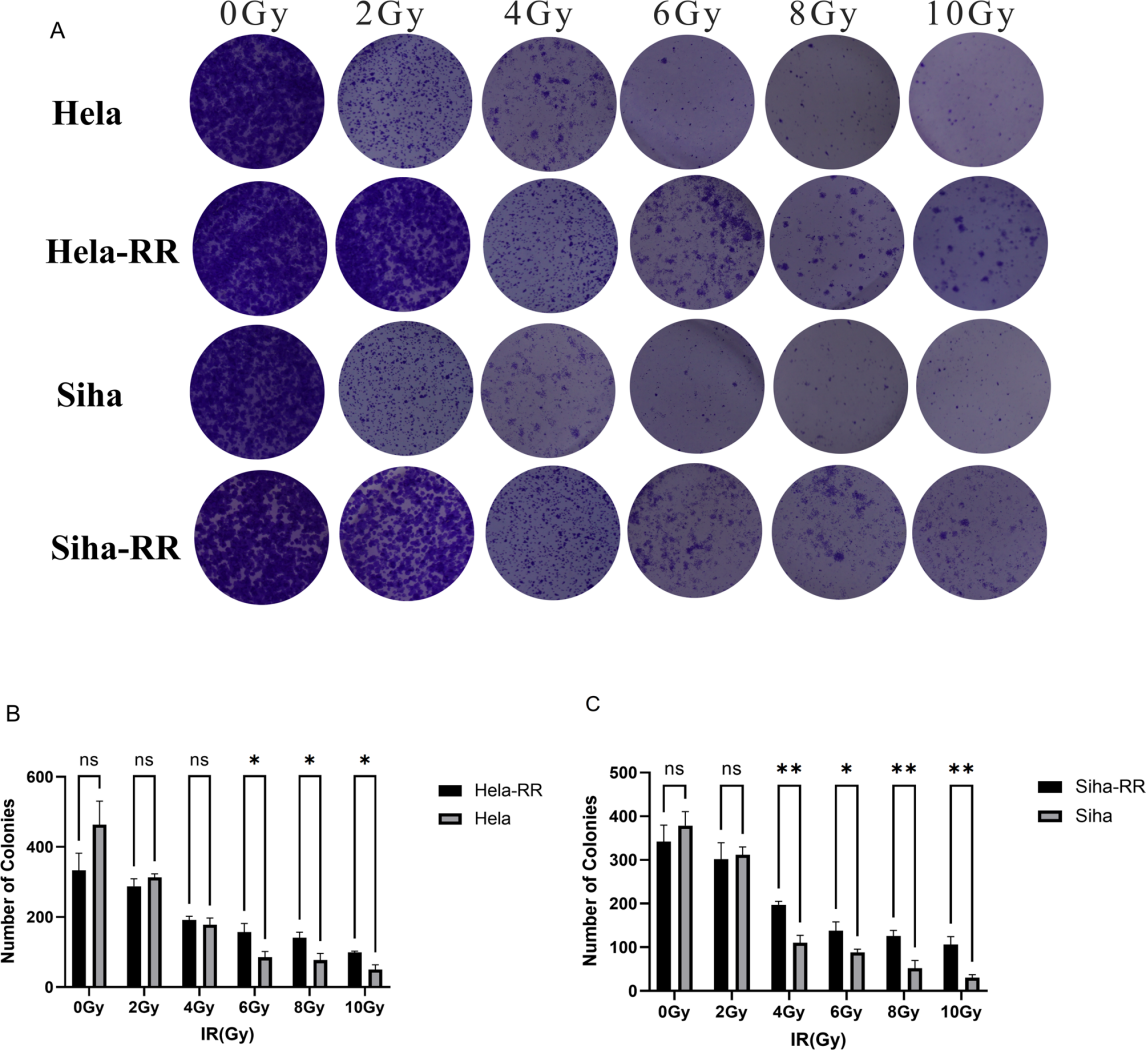
Acquired radiotherapy-resistant cell lines have enhanced clone formation after radiation exposure. **A** Representative images of colony formation assays. **B** Quantitative comparison of clonogenic capacity between Hela and Hela-RR cell lines. **C** Quantitative comparison of clonogenic capacity between Siha and Siha-RR cell lines. All data are presented as mean ± standard deviation. Statistical significance was determined by two-tailed Student’s t-test. (Significance markers: *P < 0.05, **P < 0.01, ***P < 0.001; ns = not significant (*P* > 0.05).)

To evaluate the radioresistance of the acquired radiotherapy-resistant cell lines, four cell types (HeLa, HeLa-RR, SiHa, and SiHa-RR) were subjected to a colony formation assay (Fig. 1). The results revealed that HeLa-RR and SiHa-RR cells exhibited a significantly stronger colony formation ability following irradiation compared to their corresponding parental cell lines (HeLa-RR vs. HeLa: *P* < 0.05; SiHa-RR vs. SiHa: *P* < 0.01).

### Acquired radiotherapy-resistant cell lines have enhanced cell survival after irradiation

**Fig. 2:**
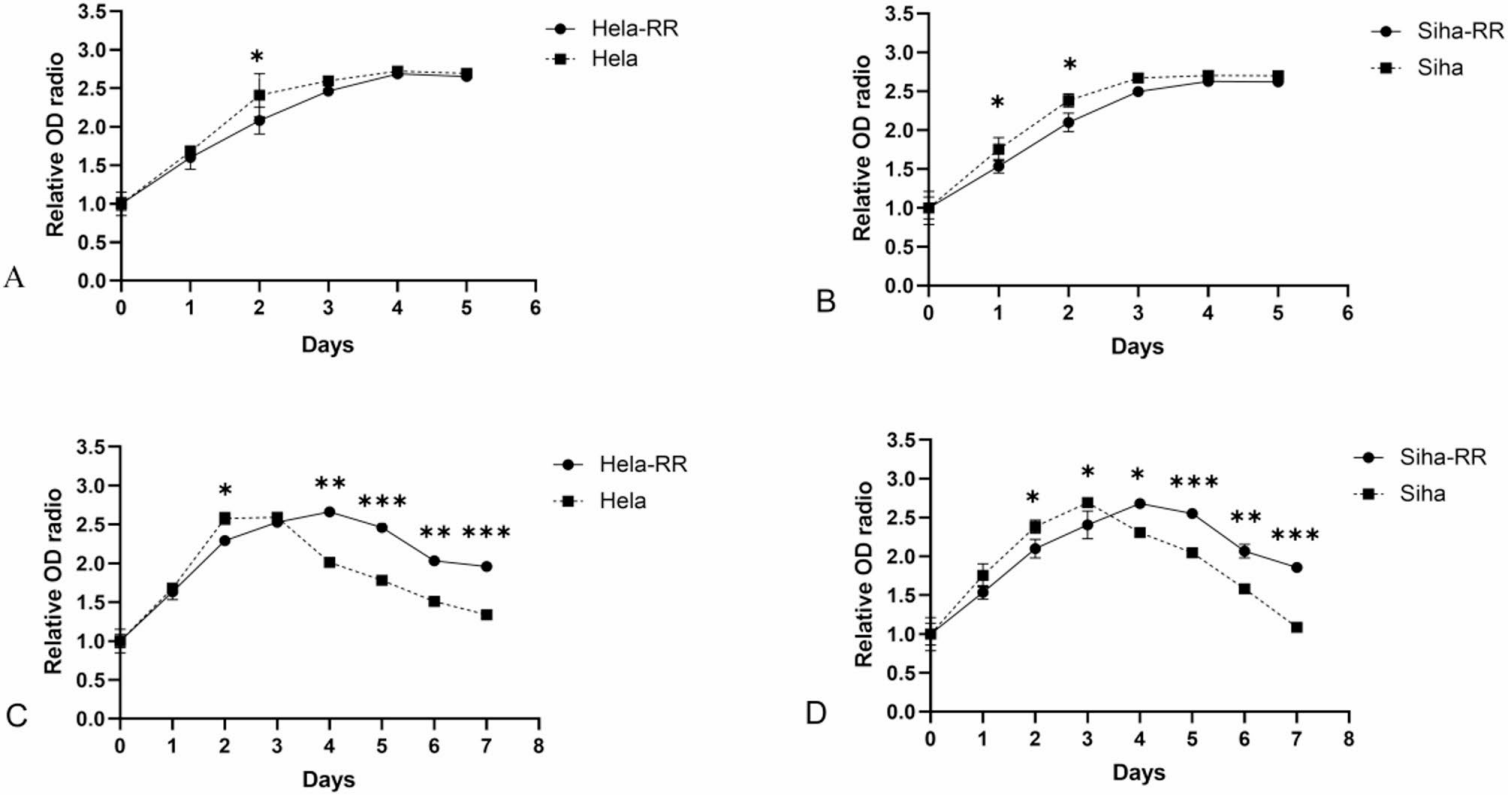
Acquired radiotherapy-resistant cell lines have enhanced cell survival after radiation exposure. **A** Time-dependent cell proliferation curves of Hela and Hela-RR cells without irradiation. **B** Time-dependent cell proliferation curves of Siha and Siha-RR cells without irradiation. **C** Time-dependent cell proliferation curves of Hela and Hela-RR cells after irradiation with a single 6 Gy dose of 6 MeV X-rays. **D** Time-dependent cell proliferation curves of Siha and Siha-RR cells after irradiation with a single 4 Gy dose of 6 MeV X-ray. All data are presented as mean ± standard deviation. Statistical significance was determined by two-tailed Student’s t-test.(Significance markers: *P < 0.05, **P < 0.01, ***P < 0.001; ns = not significant (*P* > 0.05).)

To further assess the survival of radiotherapy-resistant cell lines post-irradiation, cell proliferation assays were conducted at 24, 48, 72, and 96 h after irradiation. As shown, the proliferation of both HeLa and HeLa-RR cells decreased following 6 Gy irradiation, but the optical density (OD) value of HeLa cells decreased more rapidly than that of HeLa-RR. Similarly, a 4 Gy dose reduced the proliferation of both SiHa and SiHa-RR cells, with SiHa exhibiting a faster decrease in OD compared to SiHa-RR. These results indicate a significant increase in cell proliferation of HeLa-RR and SiHa-RR cell lines after irradiation, relative to their parental counterparts. In summary, the CCK-8 proliferation assay demonstrated that non-irradiated HeLa and SiHa cells proliferated more rapidly than their resistant counterparts, HeLa-RR and SiHa-RR (Fig. 2). Furthermore, irradiated HeLa-RR and SiHa-RR cells exhibited enhanced survival compared to the corresponding parental cell lines (HeLa-RR vs. HeLa: *P* < 0.01; SiHa-RR vs. SiHa: *P* < 0.01).

### Acquired radiotherapy-resistant cell lines are more resistant to apoptosis after irradiation

**Fig. 3:**
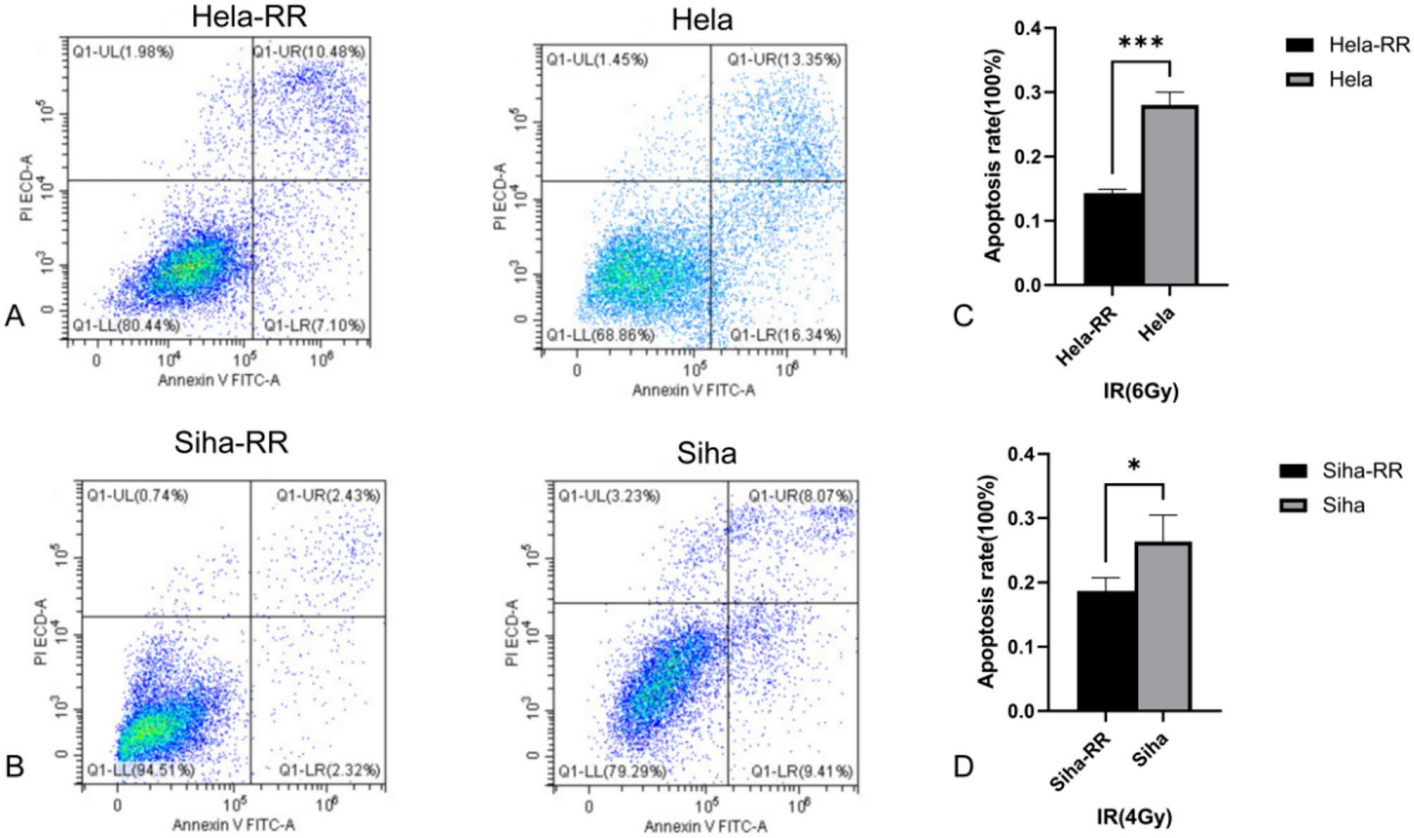
Acquired radiotherapy-resistant cell lines are more resistant to apoptosis after radiation exposure. **A** Representative images of apoptosis assay of HeLa-RR and HeLa cells 24 h post-irradiation with a single 6 Gy dose of 6 MeV X-rays. **B** Representative images of apoptosis assay of SiHa-RR and SiHa cells 24 h post-irradiation with a single 4 Gy dose of 6 MeV X-rays. **C** Quantitative comparison of resistant capacity to apoptosis after radiation exposure between HeLa-RR and HeLa cells. **D** Quantitative comparison of resistant capacity to apoptosis after radiation exposure between SiHa-RR and SiHa cells. All data are presented as mean ± standard deviation. Statistical significance was determined by two-tailed Student’s t-test.(Significance markers: *P < 0.05, **P < 0.01, ***P < 0.001; ns = not significant (*P* > 0.05).)

To evaluate the post-irradiation anti-apoptotic response of acquired radiotherapy-resistant cell lines, HeLa and HeLa-RR cells were irradiated with a single 6 Gy dose of 6 MeV X-rays, while SiHa and SiHa-RR cells were exposed to a 4 Gy dose of 6 MeV X-rays. Apoptosis was assessed 24 h post-irradiation. The results indicated that HeLa-RR and SiHa-RR cells exhibited superior anti-apoptotic abilities after X-ray exposure compared to the corresponding parental cell lines. These results suggest that the acquired radiotherapy-resistant cell lines, HeLa-RR and SiHa-RR, exhibit a significant increase in anti-apoptotic capacity post-irradiation relative to HeLa and SiHa (Fig. 3). (HeLa-RR vs. HeLa: *P* < 0.001; SiHa-RR vs. SiHa: *P* < 0.05).

### Acquired radiotherapy-resistant cell lines have a higher ratio of S phase to G2/M phase

**Fig. 4:**
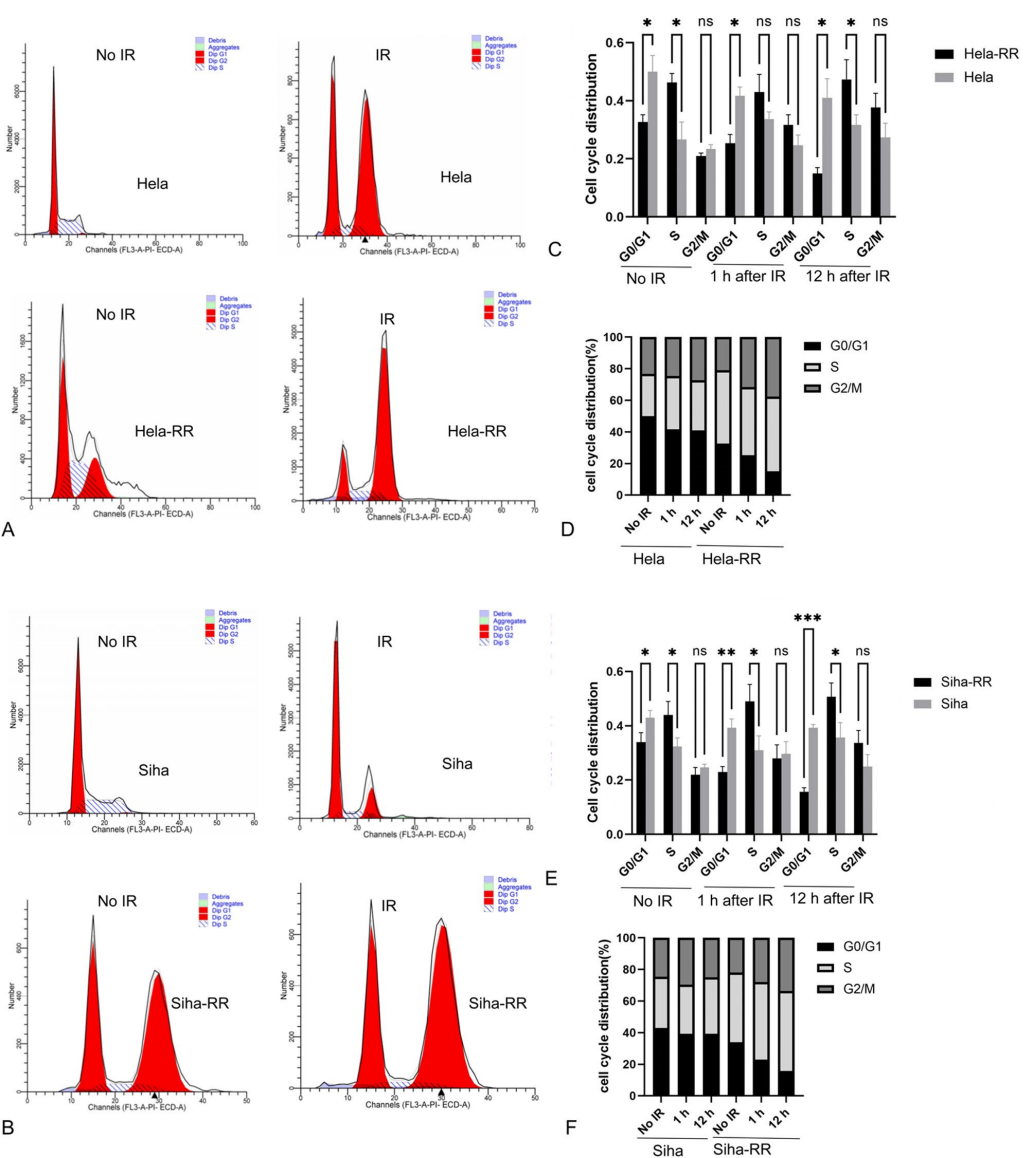
Cell cycle assay results of acquired radiotherapy-resistant cell lines corresponding parental cell lines after radiation exposure. **A** Representative images of cell cycle assay results of HeLa and HeLa-RR cells, unirradiated or 12 h post-irradiation with a single 6 Gy dose of 6 MeV X-rays. **B** Representative images of cell cycle assay results of SiHa and SiHa-RR cells, unirradiated or 12 h post-irradiation with a single 4 Gy dose of 6 MeV X-rays. **C** Quantitative comparison of G0/G1, S and G2/M phase between HeLa-RR and HeLa cells in different treatment groups (unirradiated, 1 h after irradiation, and 12 h after irradiation). **D** HeLa and HeLa-RR cells in each phase is shown in the histogram in different treatment groups. **E** Quantitative comparison of G0/G1, S and G2/M phase between SiHa-RR and SiHa cells in different treatment groups (unirradiated, 1 h after irradiation, and 12 h after irradiation). **F** HeLa and HeLa-RR cells in each phase is shown in the histogram in different treatment groups.All data are presented as mean ± standard deviation. Statistical significance was determined by two-tailed Student’s t-test.(Significance markers: *P < 0.05, **P < 0.01, ***P < 0.001; ns = not significant (*P* > 0.05).)

To evaluate the cell cycle arrest capabilities of acquired radiotherapy-resistant cell lines, HeLa-RR and SiHa-RR were compared with their respective parental cell lines, HeLa and SiHa. HeLa and HeLa-RR cells received a single 6 Gy dose of 6 MeV X-rays, while SiHa and SiHa-RR cells were irradiated with 4 Gy of 6 MeV X-rays. The cell cycle was analyzed by flow cytometry at unirradiated, 1 h after irradiation and 12 h after irradiation. Results indicated that, prior to irradiation, the proportion of cells in the S-phase and G2-phase in HeLa-RR and SiHa-RR was higher than that of the corresponding parental cell lines. After irradiation, the distribution of cells in the S-phase and G2-phase was significantly increased in HeLa-RR and SiHa-RR cells compared to the parental lines (Fig. 4). This suggests that acquired radiotherapy-resistant cell lines, HeLa-RR and SiHa-RR, exhibit a higher proportion of cells in the S-phase and G2/M-phase compared to their parental counterparts. (HeLa-RR vs. HeLa: *P* < 0.05; SiHa-RR vs. SiHa: *P* < 0.05).

### Acquired radiotherapy-resistant cell lines are more invasive after radiation exposure

**Fig. 5:**
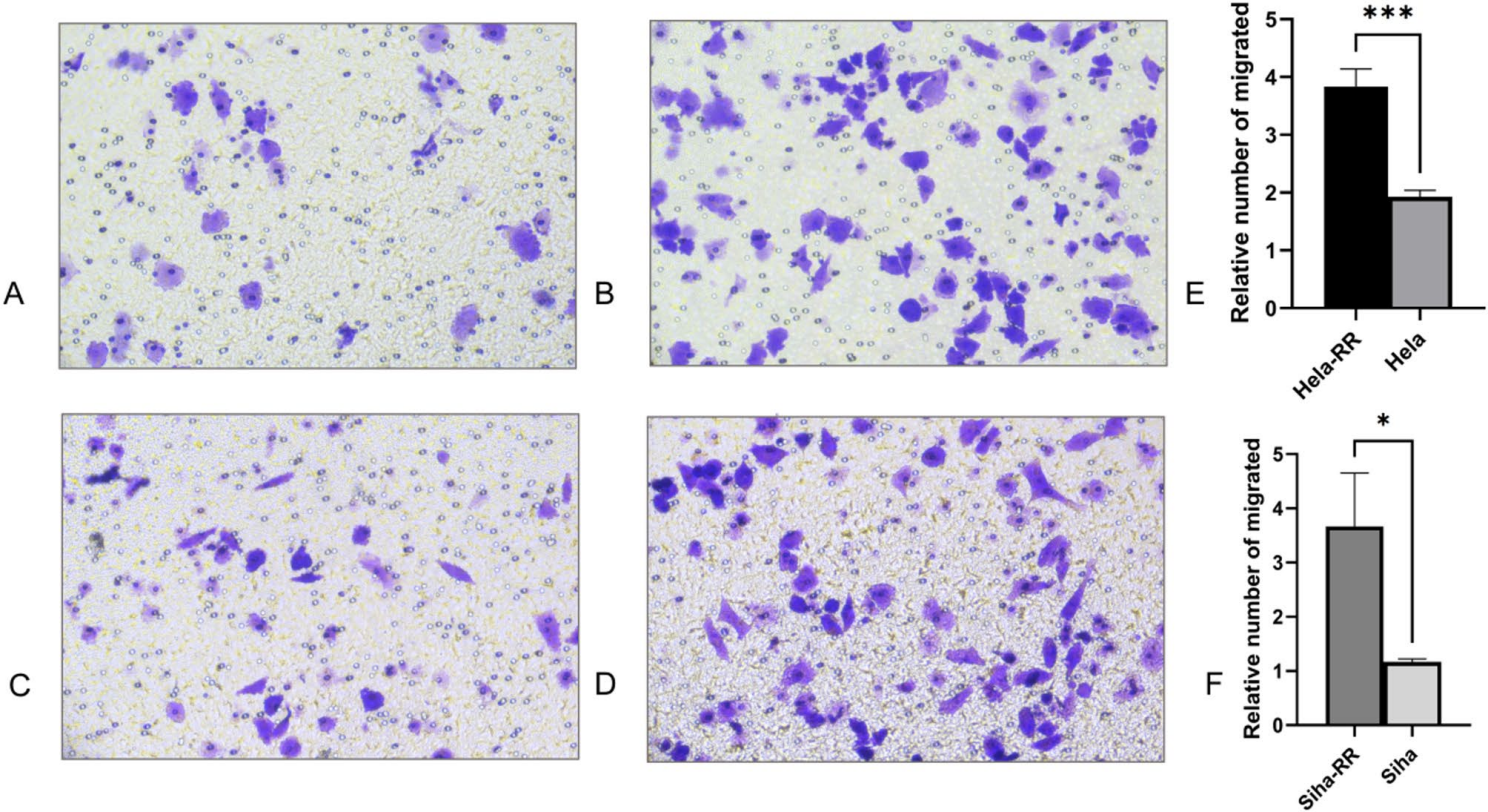
Acquired radiotherapy-resistant cell lines are more invasive after radiation exposure. **A** Representative images of cell invasion capacity assay of HeLa cells after radiation with a single 6 Gy dose of 6 MeV X-rays. **B** Representative images of cell invasion capacity assay of HeLa-RR cells after radiation with a single 6 Gy dose of 6 MeV X-rays. **C** Representative images of cell invasion capacity assay of SiHa cells after radiation with a single 4 Gy dose of 6 MeV X-rays. **D** Representative images of cell invasion capacity assay of SiHa-RR cells after radiation with a single 4 Gy dose of 6 MeV X-rays. **E** Quantitative comparison of capacity to invasive after radiation exposure between HeLa-RR and HeLa cells. **F** Quantitative comparison of capacity to invasive after radiation exposure between SiHa-RR and SiHa cells. All data are presented as mean ± standard deviation, Statistical significance was determined by two-tailed Student’s t-test. (Significance markers: *P < 0.05, **P < 0.01, ***P < 0.001; ns = not significant (*P* > 0.05).)

To assess the invasive potential of the acquired radiotherapy-resistant cell lines, invasion assays were performed following irradiation. HeLa and HeLa-RR cells were irradiated with 6 Gy of 6 MeV X-rays, and SiHa and SiHa-RR cells received 4 Gy of 6 MeV X-rays. The results (Fig. 5) revealed that HeLa-RR and SiHa-RR cells exhibited significantly greater invasive capacity after irradiation compared to their parental cell lines (HeLa-RR vs. HeLa: *P* < 0.001; SiHa-RR vs. SiHa: *P* < 0.05).

### Differential mRNA expression profiles of radiotherapy-resistant cell lines (Hela-RR vs. Hela; Siha-RR vs. Siha)

**Fig. 6:**
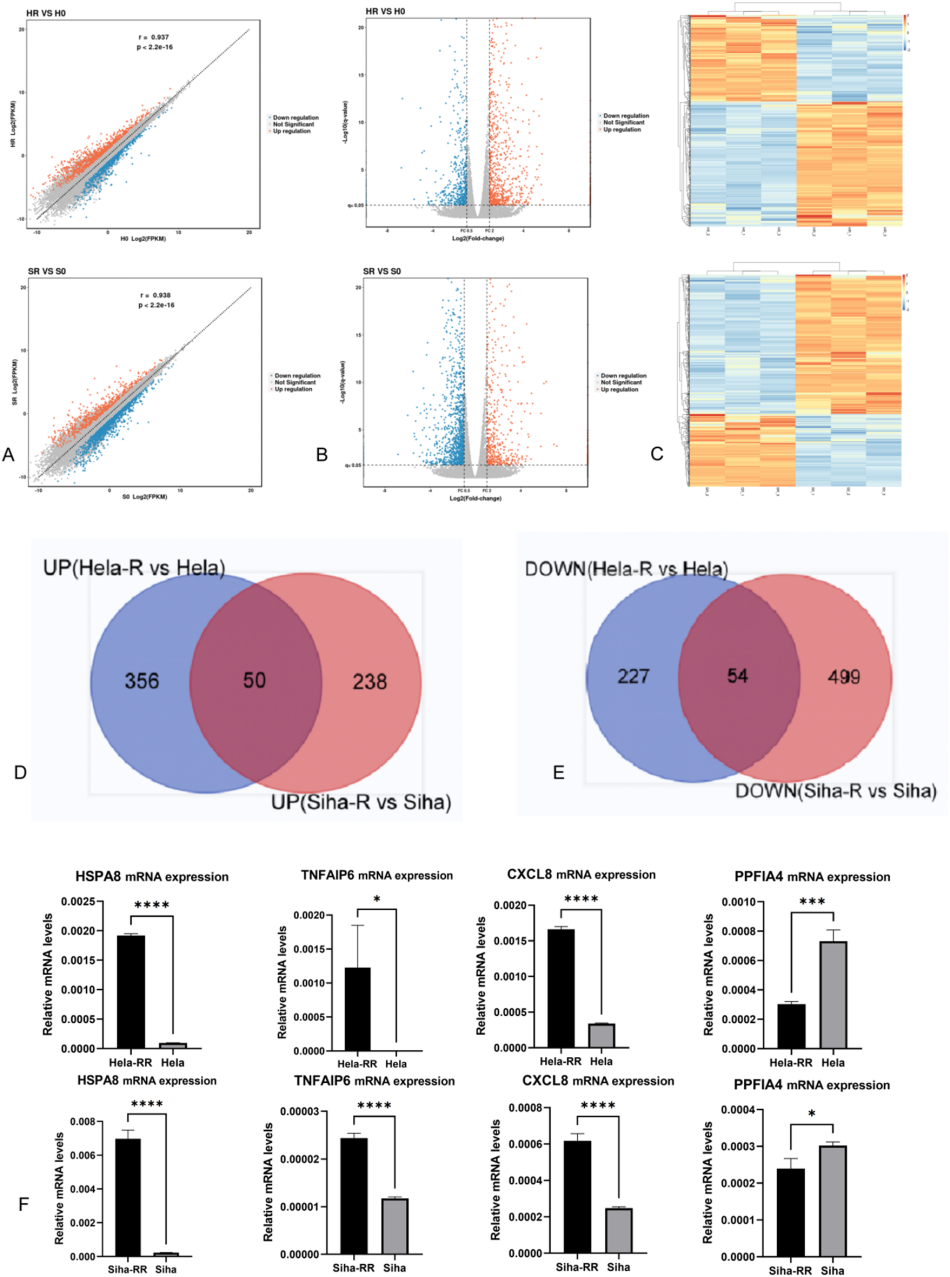
Differential mRNA expression profiles of radiotherapy-resistant cell lines compared to their parental counterparts by mRNA-seq. **A** Scatter plots of differential expression mRNA (Hela-RR vs. Hela; Siha-RR vs. Siha). **B** Volcano plots of differential expression mRNA (Hela-RR vs. Hela; Siha-RR vs. Siha). **C** Hotspot plots of differential expression mRNA (Hela-RR vs. Hela; Siha-RR vs. Siha). **D** Venn diagram displaying the differential up-regulated mRNAs of two cervical cancer acquired radiotherapy resistant cell lines compared to their respective corresponding parental cell lines. **E** Venn diagram displaying the differential down-regulated mRNAs of two cervical cancer acquired radiotherapy resistant cell lines compared to their respective corresponding parental cell lines. **F** Quantitative comparison of qRT-PCR result of mRNA differential levels of HSPA8, TNFAIP6, CXCL8, PPFIA4 in Hela-RR and Hela, Siha-RR and Siha cells. Statistical significance was determined by two-tailed Student’s t-test. (Significance markers: *P < 0.05, **P < 0.01, ***P < 0.001; ****P < 0.0001; ns = not significant (*P* > 0.05).)

The quality control of Hela-RR, Hela, Siha-RR and Siha cells which were subjected to high-throughput mRNA sequencing (mRNA-seq) was satisfactory (Supplementary Table 3).). The mRNA differential expression profiles of acquired radiotherapy-resistant CC cell lines, HeLa-RR and SiHa-RR, were mapped using mRNA-seq, as shown in the scatter plots, volcano plots, and hotspot plots (Fig. 6A, B &C). The comparison revealed that in HeLa-RR cells, 406 mRNAs were upregulated, while 281 mRNAs showed decreased expression. In SiHa-RR cells, 288 mRNAs were upregulated, and 553 mRNAs were downregulated compared to their respective parental cell lines (Supplementary Table 4). Gene Ontology (GO) and Kyoto Encyclopedia of Genes and Genomes (KEGG) enrichment analyses of the differentially expressed genes indicated that DNA-related biological processes, signaling molecules and their interactions, the immune environment, and various signaling pathways play critical roles in the mechanism underlying radiotherapy resistance in CC cells (Supplementary Fig. 3). The differential mRNA expression patterns in HeLa-RR and SiHa-RR cells, compared to their parental counterparts, were further analyzed by Venn diagram plots. The intersection of the upregulated and downregulated mRNAs between the two cell lines revealed 50 upregulated and 54 downregulated genes common to both HeLa-RR and SiHa-RR cells (Fig. 6D, E). The top 10 most significant differential genes identified were IL11, CXCL8, MMP1, HSPA8, CA9, PPFIA4, EDN2, GUCY1A2, EFNA3, and TNFAIP6 ( Supplementary Table 5). To validate the accuracy of the mRNA-seq results obtained using flux sequencing technology, qRT-PCR was performed to verify the differential expression levels of HSPA8, TNFAIP6, CXCL8, and PPFIA4 in the HeLa-RR and HeLa cells, as well as SiHa-RR and SiHa cells. The results (Fig. 6F), demonstrated that the expression trends were consistent with those observed in the mRNA-seq analysis, confirming the reliability of the high-throughput sequencing results.

### Differential expression of CRRF-associated prognostic genes in cervical cancer

**Fig. 7:**
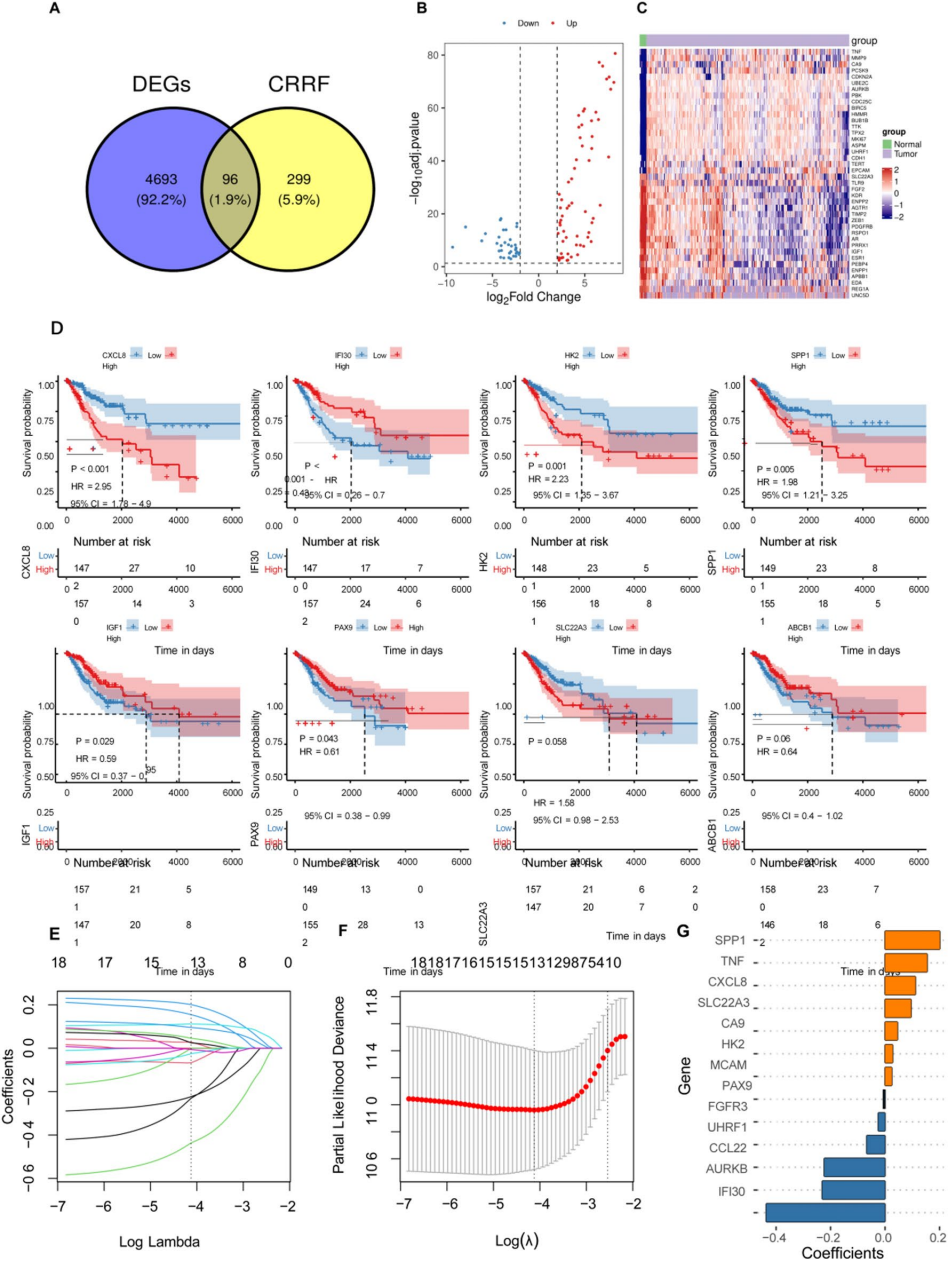
Differential expression of CRRF-associated prognostic genes in cervical cancer. **A** Venn maps for taking differential expression gene intersections with CRRFs by using the TCGA-CESC dataset. **B** Volcano plots of differential expression gene by using the TCGA-CESC dataset with a threshold filter of FDR < 0.05 and |log2FC| > 2. **C** Heat maps of differential expression gene by using the TCGA-CESC dataset with a threshold filter of FDR < 0.05 and |log2FC| > 2. **D** KM curves for the one-factor cox significant factor top8: CXCL8, IFI30, HK2, SPP1, IGF1, PAX9, SLC22A3, and ABCB1. **E**–**G** Lasso-cox analysis to construct the model: **E** Trajectory of change of each independent variable, where the log value of the independent variable lambda is shown on the horizontal axis and the coefficients of the independent variables are shown on the vertical axis. **F** Confidence intervals under each lambda. **G** Regression coefficients of Signature.R (4.2.1) were used for statistics and visualization. Kaplan–Meier analysis, log-rank test and Cox’s proportional hazards regression model were applied to analyze the corresponding data. The combination score was determined using SynergyFinder. (Significance markers: *P < 0.05, **P < 0.01, ***P < 0.001; ****P < 0.0001; ns = not significant (*P* > 0.05).)

Differential expression analysis was then conducted using the TCGA-CESC dataset with a threshold filter of FDR < 0.05 and |log2FC| > 2. This analysis identified a total of 4789 differentially expressed genes, comprising 3368 downregulated and 1421 upregulated genes. These genes were intersected with CRRFs, yielding 96 differentially expressed CRRFs (Fig. 7A). The volcano plot and heatmap of the differentially expressed genes (Fig. 7B, C), with the heatmap highlighting the top 40 CRRF genes based on their absolute log2FC values. GO and KEGG enrichment analyses of the CRRF-related differentially expressed genes indicated that cell cycle, Oocyte meiosis and various signaling pathways play critical roles in the mechanism (Supplementary Fig. 4). Using the 96 differentially expressed CRRFs, one-way Cox regression analysis was performed to identify prognostic factors with a P-value less than 0.05, resulting in 18 significant prognostic factors. Among these, 8 genes with the most significant P-values (CXCL8, IFI30, HK2, SPP1, IGF1, PAX9, SLC22A3, and ABCB1) were selected to generate Kaplan-Meier (KM) curves for survival analysis (Fig. 7D). Redundant genes were excluded using LASSO-Cox, leading to the identification of 13 key genes to construct the prognostic model (Fig. 7E–G). Clinical information statistics of TCGA training set cohort are presented in Supplementary Table 6.

### CXCL8 expression is higher in cervical cancer than in adjacent normal tissues

**Fig. 8:**
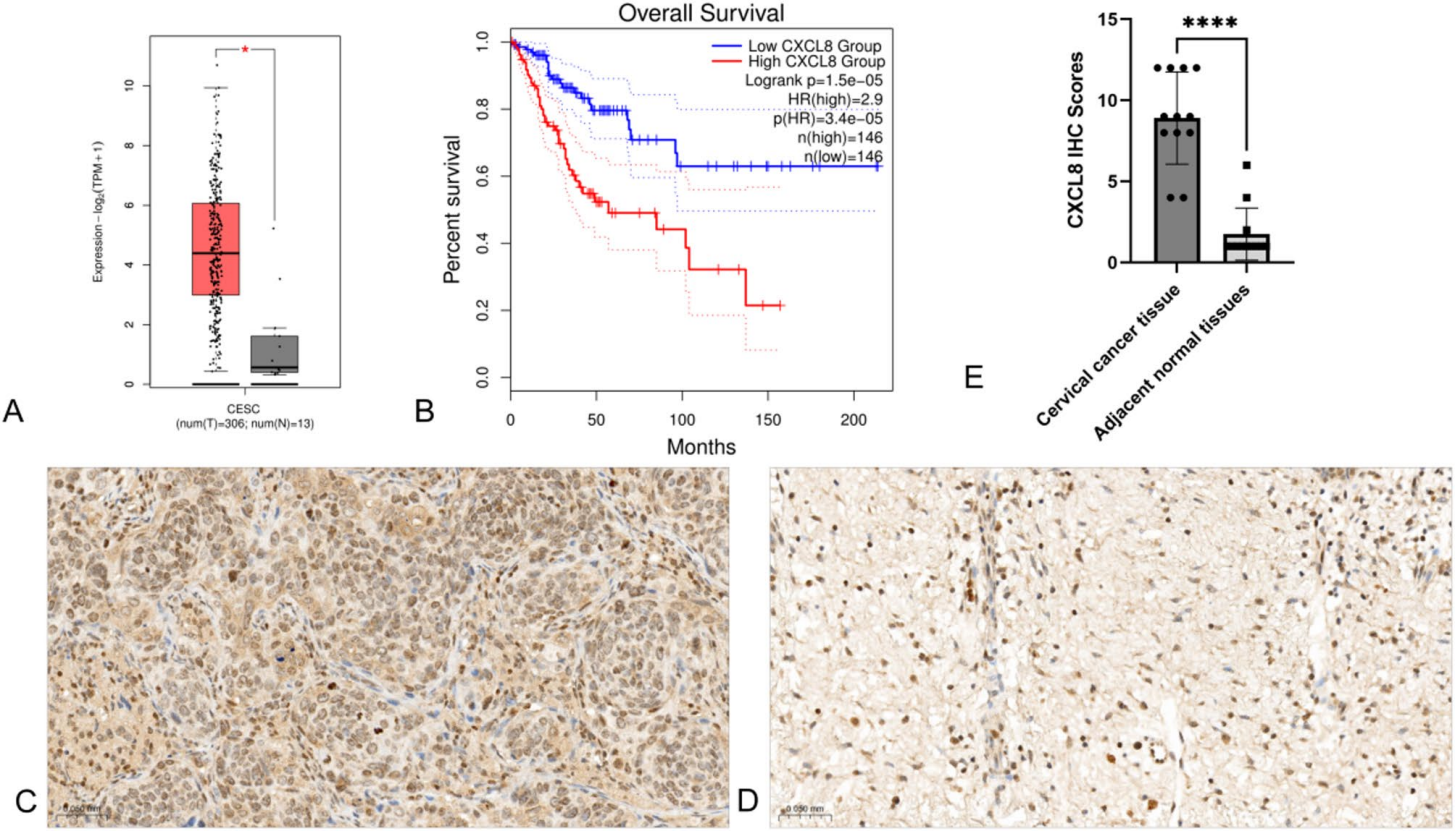
CXCL8 expression is higher in cervical cancer than in adjacent normal tissues by GEPIA2 analysis and immunohistochemical verification. **A** CXCL8 expression in cervical cancer by GEPIA2 analysis. **B** Survival analysis of CXCL8 expression in cervical cancer by GEPIA2 analysis. **C** Immunohistochemical verification of CXCL8 expression in cervical cancer tissues. **D** Immunohistochemical verification of CXCL8 expression in adjacent normal tissues. **E** The expression of CXCL8 in tumor tissues from cervical cancer patients and corresponding adjacent normal tissue was examined by immunohistochemical analysis (*n* = 12). All 12 paired IHC images for CXCL8 expression are presented in Supplementary Fig. 5. Statistical significance of CXCL8 expression in cervical cancer tissues and corresponding adjacent normal tissue was determined by paired t-test. (Significance markers: *P < 0.05, **P < 0.01, ***P < 0.001; ****P < 0.0001; ns = not significant (*P* > 0.05).)

Analysis using GEPIA2, in conjunction with large datasets from TCGA and GTEx, reveals that CXCL8 expression is significantly elevated in CC tissues compared to normal tissues. Furthermore, survival analysis indicates that high CXCL8 expression correlates with poorer prognosis in patients with CC. Subsequent immunohistochemical analysis of 12 paired CC and adjacent normal tissues confirmed that CXCL8 expression was higher in tumor tissues, consistent with GEPIA2 findings (Fig. 8).

### In vitro knockdown of CXCL8 knockdown restores radiotherapy sensitivity in acquired radioresistant cervical cancer cell lines

**Fig. 9:**
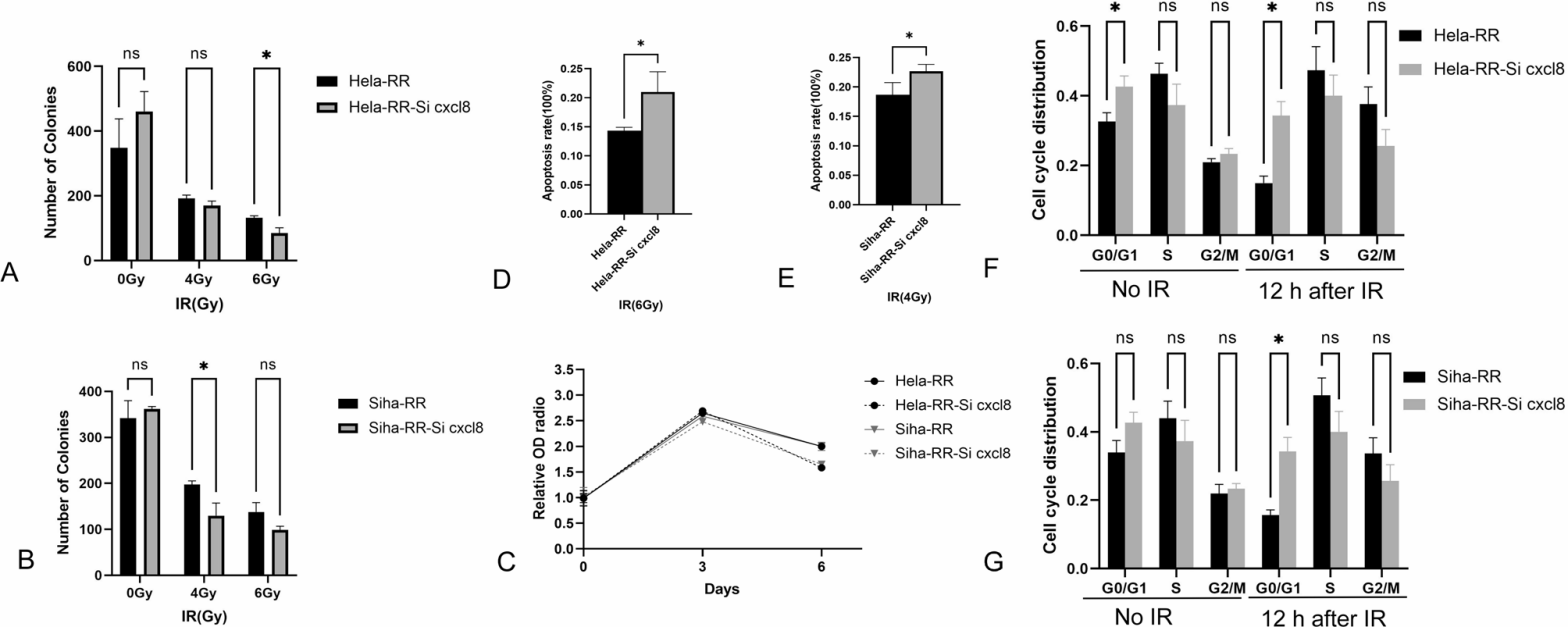
CXCL8 knockdown restores radiotherapy sensitivity in acquired radioresistant cervical cancer cell lines. **A** Quantitative comparison of clonogenic capacity of Hela-RR cell lines with or without CXCL8 knockdown after irradiation. **B** Quantitative comparison of clonogenic capacity of Siha-RR cell lines with or without CXCL8 knockdown after irradiation. **C** Time-dependent cell proliferation curves of Hela-RR and Siha-RR cells with or without CXCL8 knockdown after irradiation. **D** Quantitative comparison of resistant capacity to apoptosis after radiation exposure with or without CXCL8 knockdown in Hela-RR lines. **E** Quantitative comparison of resistant capacity to apoptosis after radiation exposure with or without CXCL8 knockdown in Siha-RR lines. **F** Quantitative comparison of G0/G1, S and G2/M phase with or without CXCL8 knockdown in Hela-RR lines in different treatment groups (unirradiated and 12 h after irradiation). **G** Quantitative comparison of G0/G1, S and G2/M phase with or without CXCL8 knockdown in Siha-RR cells in different treatment groups (unirradiated and 12 h after irradiation).All data are presented as mean ± standard deviation. Statistical significance was determined by two-tailed Student’s t-test.(Significance markers: *P < 0.05, **P < 0.01, ***P < 0.001; ns = not significant (*P* > 0.05).)

CXCL8 RNA levels were assessed by qRT-PCR 48 h post-transfection with SiCXCL8 and the results indicate a significant reduction in CXCL8 RNA expression in acquired radioresistant CC cell lines after transfection, confirming the success of the transfection process (Supplementary Fig. 6). To examine the impact of CXCL8 knockdown on the radiotherapy sensitivity of these acquired radioresistant CC cell lines, a series of assays—including cell viability, colony formation, apoptosis, and cell cycle analysis—were conducted on Hela-RR and Siha-RR cells transiently transfected with SiCXCL8 (Fig. 9). CXCL8 knockdown in these radioresistant cell lines led to reduced cell proliferation, decreased colony formation, an increased S-phase and G2/M-phase ratio, and enhanced apoptosis (*P* < 0.05), indicating that CXCL8 silencing restored radiosensitivity in the acquired radioresistant CC cell lines.

### In vitro exogenous CXCL8 promotes radiotherapy resistance in parental cell lines

**Fig. 10:**
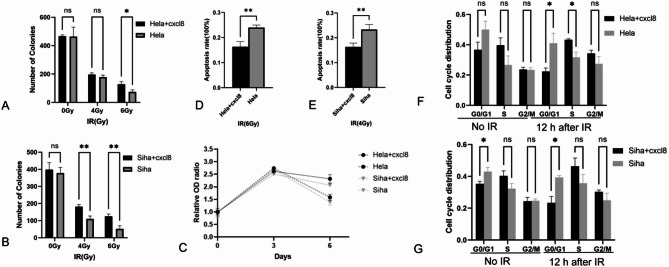
Exogenous CXCL8 induces radiotherapy resistance in parental cell lines. **A** Quantitative comparison of clonogenic capacity of Hela cell lines with or without exogenous CXCL8 after irradiation. **B** Quantitative comparison of clonogenic capacity of Siha cell lines with or without exogenous CXCL8 after irradiation. **C** Time-dependent cell proliferation curves of Hela and Siha cells with or without exogenous CXCL8 after irradiation. **D** Quantitative comparison of resistant capacity to apoptosis after radiation exposure with or without exogenous CXCL8 in Hela lines. **E** Quantitative comparison of resistant capacity to apoptosis after radiation exposure with or without exogenous CXCL8 in Siha lines. **F** Quantitative comparison of G0/G1, S and G2/M phase with or without exogenous CXCL8 in Hela lines in different treatment groups (unirradiated and 12 h after irradiation). **G** Quantitative comparison of G0/G1, S and G2/M phase with or without exogenous CXCL8 in Siha cells in different treatment groups (unirradiated and 12 h after irradiation).All data are presented as mean ± standard deviation. Statistical significance was determined by two-tailed Student’s t-test.(Significance markers: *P < 0.05, **P < 0.01, ***P < 0.001; ns = not significant (*P* > 0.05).)

To assess the impact of exogenous CXCL8 on the radiotherapy sensitivity of parental CC cell lines, Hela and Siha cells were treated with exogenous CXCL8 (100 ng/mL final concentration), and a similar set of assays were performed 48 h post-treatment. Exogenous CXCL8 enhanced cell proliferation, increased colony formation, decreased S-phase and G2/M-phase ratios, and inhibited apoptosis, suggesting that the addition of CXCL8 confers resistance to radiotherapy in these parental cell lines (Fig. 10).

## Discussion

Cervical cancer remains a leading cause of cancer-related mortality in women globally^26^, despite advances in screening^27^, HPV vaccination^28,29^, and radiotherapy techniques such as cisplatin-based chemoradiotherapy, intensity-modulated radiation, and image-guided brachytherapy^30^. Persistent challenges include suboptimal 5-year survival rates, 20–65% for locally advanced stages^31^, driven by tumor recurrence, metastasis, and radioresistance, underscoring the need for novel therapeutic strategies to enhance treatment efficacy^9^.

Radiotherapy resistance, mediated by cancer cells’ ability to evade radiation-induced oxidative stress^32^ and apoptosis, drives tumor recurrence, metastasis^33^, and poor clinical outcomes^34^. This resistance encompasses intrinsic heterogeneity in radiosensitivity and acquired adaptations to treatment^35^, collectively limiting therapeutic efficacy in advanced cancers^36^. Radioresistance in CC involves multifactorial mechanisms including hypoxia-mediated Hypoxic Inducible Factor (HIF) activation (HIF-1α/2α/3α)^37^, enhanced DNA damage repair via Ataxia Telangiectasia Mutated (ATM)/p53 signaling^38^ and double-strand breaks (DSBs) repair pathways (Non-Homologous End Joining (NHEJ) and Homologous Recombination (HR))^39^, and apoptosis evasion^40^. TME interactions^41^, cancer stem cells^42^, and epigenetic regulators further contribute to resistance^43^. While positive HIF-2αcorrelates with radiotherapy failure and local recurrence^44^, HIF-3α overexpression promotes radioresistance in female rectal cancer^45^. Dysregulation of these pathways underscores the biological complexity of treatment resistance, necessitating mechanistic studies to identify actionable targets for radiosensitization.

Standardized radioresistant CC cell models remain unestablished due to prolonged maintenance challenges post-irradiation^46^. Radioresistant cervical cancer cell lines (Hela-RR/Siha-RR) were established via intermittent fractionated irradiation, thereby inducing acquired radioresistance in the parental CC cell lines^47^, mimicking clinical protocols for nearly a year, overcoming limitations of single-dose approaches^48^. Colony formation assays demonstrated enhanced clonogenicity in resistant lines, while post-irradiation proliferation assays revealed delayed growth arrest compared to parental cells. Cell cycle analysis showed Hela-RR and Siha-RR increased S-phase without irradiation. Cell cycle analysis showed resistant lines exhibited increased S-phase populations without irradiation, with increased S/G2-M phases post-treatment. Radiobiological characterization^49^ indicated that Hela-RR and Siha-RR cell exhibited some degree of radiotherapy resistance, compared to parental cell lines Hela and Siha. At the same time, our results also showed that the acquired radiotherapy resistant cell lines were more resistant to apoptosis and invasive than the parent cell lines after irradiation. This design ensured that the observed phenotypic differences between the parental and resistant cell lines could be attributed solely to molecular-level variations, providing a solid foundation for further experimental investigations.

The TME, composed of the extracellular matrix (ECM), immune cells, and stromal cells^50^, undergoes continuous modification due to the proliferation of tumor cells^51^. The TME in cervical cancer drives radioresistance through hypoxia^52^, metabolic reprogramming^53^, and epithelial-mesenchymal transition (EMT) activation^54^, which promote cancer stemness and immune evasion. These transcription factors trigger signaling pathways that reinforce the ability of tumor cells to undergo EMT, thereby fostering radiotherapy resistance. Moreover, EMT-induced transcription factors bestow CSC properties on tumor cells and facilitate CSC production^55^. Tumor cells exposed to radiation can release a variety of immune-related molecules, including tumor-associated antigens, chemokines, and inflammatory mediators. Chemokines, with their dual tumor-promoting and anti-tumor effects, have a complex role in the response to radiotherapy. Targeting specific chemokines may attenuate radiation damage or enhance tumor control, depending on their influence on the radiotherapy response^56^.

High-throughput mRNA sequencing^57^ of radioresistant cervical cancer cell lines (Hela-RR/Siha-RR) and parental counterparts identified 687 and 841 differentially expressed mRNAs, respectively, with 50 common dysregulated genes across both resistant models. KEGG pathway analysis (http://www.kegg.jp/ or http://www.genome.jp/ kegg) highlighted pathways linked to DNA repair, hypoxia, and the TME^57^. The most significant genes that were commonly upregulated and downregulated in both Hela-RR and Siha-RR cells included IL11, CXCL8, MMP1, HSPA8, CA9, PPFIA4, EDN2, GUCY1A2, EFNA3, and TNFAIP6. A study by Sun et al. found that IL-11 levels were significantly upregulated in radio-resistant CC cells and that IL-11 conferred radioresistance through activation of the PI3K/Akt signaling pathway, suggesting that IL-11 may play a role in radioresistance and could serve as an effective target for radiosensitization in CC treatment^58^. CXCL8 has been shown to be significantly overexpressed in various cancers, including prostate, colorectal, and squamous cell carcinomas of the head and neck^22,23^. Additionally, CXCL8 has been implicated in promoting resistance to chemotherapy, molecular targeted therapy, and immune checkpoint inhibition (ICI) therapy^23,59^. High expression of MMP1 and HSPA8 has been associated with poor prognosis in various malignancies^60^, and the findings from the current study align with these previous reports. The qRT-PCR confirmed consistent expression trends for TNFAIP6, CXCL8, HSPA8, and PPFIA4, aligning with sequencing data. These findings establish CXCL8 as a novel mediator of acquired radioresistance in cervical cancer, supported by cross-validation and pathway coherence, providing a molecular foundation for targeted radiosensitization strategies.

Tumor radiotherapy sensitivity is crucial for precision treatment, with resistance often linked to genetic/phenotypic changes post-radiation^61^. To address knowledge gaps, Wen et al. developed the Database of Cancer Radiation Sensitivity Regulators (dbCRSR) (URL:http://bioinfo.ahu.edu.cn:8080/dbCRSR), the first database cataloging 820 radiosensitivity modifiers^62^. In this study, differential expression analysis of TCGA-CESC data (FDR < 0.05, |log2FC|>2) identified 4,789 DEGs, intersecting with cancer radiation response factors (CRRFs) to yield 96 differentially expressed CRRFs. Functional enrichment revealed cell cycle/proliferation pathways. One-way Cox regression identified 18 prognostic factors, with eight key genes (CXCL8, IFI30, HK2, SPP1, IGF1, PAX9, SLC22A3, ABCB1) validated via KM analysis. LASSO-Cox regression refined these to 13 critical genes for prognostic modeling. The validated model demonstrated independent prognostic capability, highlighting its feasibility for guiding therapeutic strategies in cervical cancer radiotherapy. This integrative approach bridges molecular mechanisms with clinical outcomes, advancing personalized radioresistance management.

mRNA profiling and TCGA-CESC analysis identified CXCL8 as a key radioresistance mediator in cervical cancer via prognostic modeling. As a pro-tumor chemokine, CXCL8 plays a pivotal role in the progression, prognosis, and drug resistance across various cancers. However, its involvement in radiotherapy resistance in CC remains unexplored. Utilizing GEPIA2 and integrating large datasets from TCGA and GTEx, our analysis revealed that CXCL8 expression is significantly higher in CC tissues compared to normal tissues. Survival analysis further demonstrated that high CXCL8 expression correlates with poorer prognosis in patients with CC. Immunohistochemical staining of 12 pairs of CC and adjacent non-cancerous tissues confirmed these findings, showing elevated CXCL8 expression in cancerous tissues. These results not only corroborate the GEPIA2 data but also reinforce the potential of CXCL8 as a promising radiosensitization target for CC therapy.

CXCL8, a pro-inflammatory chemokine, binds CXCR1/CXCR2 receptors to drive tumor progression via TME interactions^24,63^. Produced by tumor/stromal/immune cells^21^, it promotes tumor proliferation, EMT, angiogenesis, and immunosuppression. Elevated in cancers, such as breast, prostate and gliomas, CXCL8 correlates with advanced stages, poor survival, and therapy resistance^22–25^. It recruits tumor-associated neutrophils (TANs)^64^, particularly pro-tumor N2 phenotypes^65^, fostering immune evasion^66^. An autocrine loop between CXCL8 and EMT amplifies cytokine cascades, enhancing metastatic potential^67^. In this study, CXCL8 emerged as a critical mediator of radiotherapy resistance in cervical cancer, implicating its role in acquired radioresistance. Targeting CXCL8-CXCR1/2 signaling may disrupt TME-driven resistance mechanisms, positioning CXCL8 as a promising therapeutic target for radiosensitization in CC.

Our study demonstrated that CXCL8 knockdown in HeLa-RR/SiHa-RR cells reduced proliferation, colony formation, and induced G2/M arrest and apoptosis, enhancing radiosensitivity. Conversely, exogenous overexpression of CXCL8 in parental HeLa/SiHa cells promoted proliferation, colony growth, and reduced apoptosis, conferring radioresistance. These findings align with studies linking CXCL8 to radiotherapy outcomes: Thomass^68^ associated CXCL8-dependent NK cell activity with improved survival post-radiotherapy, while Sun implicated IL-11/PI3K/Akt signaling in CC radioresistance^56^. While this study similarly explored the effects of cytokines on radiosensitization, our approach differs in that the increase of exogenous CXCL8 was inspired by Jiang et al.^69^, who used CXCL8 to stimulate the human osteosarcoma cell line MG-63, analyzing its effects on invasion, proliferation, apoptosis, and associated signaling pathways. These studies suggest that the discovery of additional cytokines and chemokines as emerging predictors of therapeutic efficacy or sensitization targets in tumor therapy is inevitable. Further exploration of cytokines like CXCL8 may unveil novel therapeutic strategies to overcome radioresistance in CC.

Sun implicated that IL-11 high expression is associated with cervical cancer cell radioresistance with 6 Gy X-ray irradiation for HeLa, SiHa, CaSki and C33A cells^56^.While in our study, radiation dosing decisions ( 6 Gy for HeLa and HeLa-RR,4 Gy for Siha and Siha-RR) based on Sublethal Single-Dose Irradiation assay in Methods section. The 6 Gy cumulative dose for HeLa-RR and 4 Gy cumulative dose for Siha-RR establishment reflects in vitro models of acquired resistance, mirroring prior studies^70^ and approximating total doses used in brachytherapy boost protocols^71^ .Dose selection aligns with clinically relevant fractionated radiotherapy regimens for cervical cancer.

This study established CC radioresistant cell lines via clinically relevant irradiation, identified CXCL8 as a key regulator through mRNA sequencing and Venn analysis of radiosensitivity-related genes. CXCL8 knockdown sensitized resistant cells to radiation, while exogenous CXCL8 induced resistance in parental lines. While CXCL8 is known to promote metastasis in other cancers, our study is the first to demonstrate its necessity for acquired radioresistance in cervical cancer via in vitro and clinical cohort validation. Unlike prior reports linking CXCL8 to chemotherapy resistance, our data reveal its unique regulation in acquired radiotherapy-resistant cell lines HeLa-RR and Siha-RR. Current limitations include unresolved CXCL8 interactions with other radiosensitivity regulators and absence of patient-derived xenograft (PDX) models. Future studies will integrate multi-omics approaches, PDX validation, and CRISPR-mediated mechanistic dissection to advance CXCL8 as a radiosensitizing chemokine target, with ongoing preclinical efforts aimed at overcoming clinical radioresistance.

## Electronic supplementary material

Below is the link to the electronic supplementary material.

Supplementary Material 1

Supplementary Material 2

Supplementary Material 3

Supplementary Material 4

Supplementary Material 5

Supplementary Material 6

Supplementary Material 7

Supplementary Material 8

Supplementary Material 9

Supplementary Material 10

Supplementary Material 11

Supplementary Material 12

## Data Availability

Clinical information and expression data of cervical cancer were obtained from the TCGA-GTEx-CESC dataset(https://xenabrowser.net/datapages/).Scoring system was validated using the GPL6244 platform GSE52903 dataset based on the GEO database (https://www.ncbi.nlm.nih.gov/geo/). R version(4.2.1) were used for some statistics (https://www.R-project.org).The database of cancer radiation sensitivity regulators in this study are available at dbCRSR database (URL: http://bioinfo.ahu.edu.cn:8080/dbCRSR/).All the other data that support the findings of this study are available from the corresponding author upon reasonable request.

## Abbreviations

AUC: Area under the ROC curve
CC: Cervical cancer
CRRF: Cancer radiosensitivity regulation factor
CSC: Cancer stem cell
CXCL8: C-X-C motif chemokine ligand 8
CXCR: C-X-C motif chemokine receptor
DDR: DNA damage response
EMT: Epithelial–mesenchymal transition
GEPIA: Gene expression profiling interactive analysis
HIF: Hypoxic inducible factor
HR: Homologous recombination
ICI: Immune checkpointInhibitors
LACC: Locally advanced cervical cancer
NHEJ: Nonhomologous end-joining
PDX: Patient-derived xenograft
RR: Radiotherapy resistance
RT: Radiotherapy
TAM: Tumor-associated macrophages
TAN: Tumor-associated neutrophil
TCGA: The Cancer Genome Atlas
TME: Tumor microenvironment

## Declarations

### Registry and registration number

N/A.

### Animal studies

N/A.
