# Supplementary figures and images for: CXCL8 is essential for cervical cancer cell acquired radioresistance and acts as a promising therapeutic target in cervical cancer
Source: Sci Rep. 2025 Jul 1;15:20467. doi: 10.1038/s41598-025-05435-w (PMC12219856; doi:10.1038/s41598-025-05435-w)

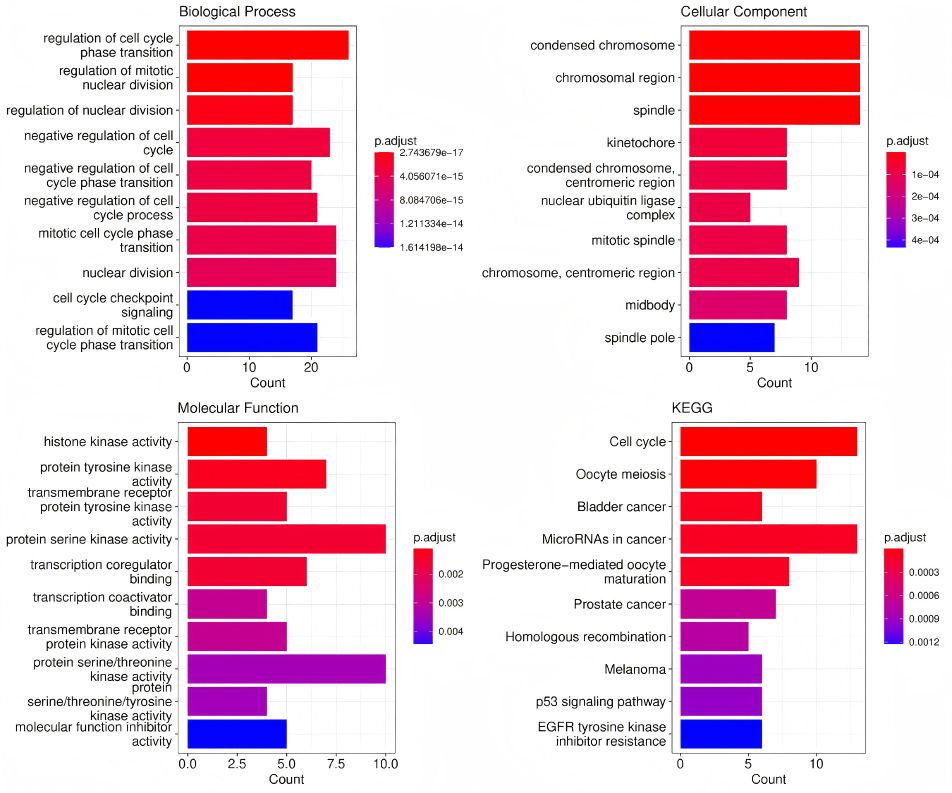

Supplement: Supplementary file 5 — Supplementary Material 5 [file 41598_2025_5435_MOESM5_ESM.png]

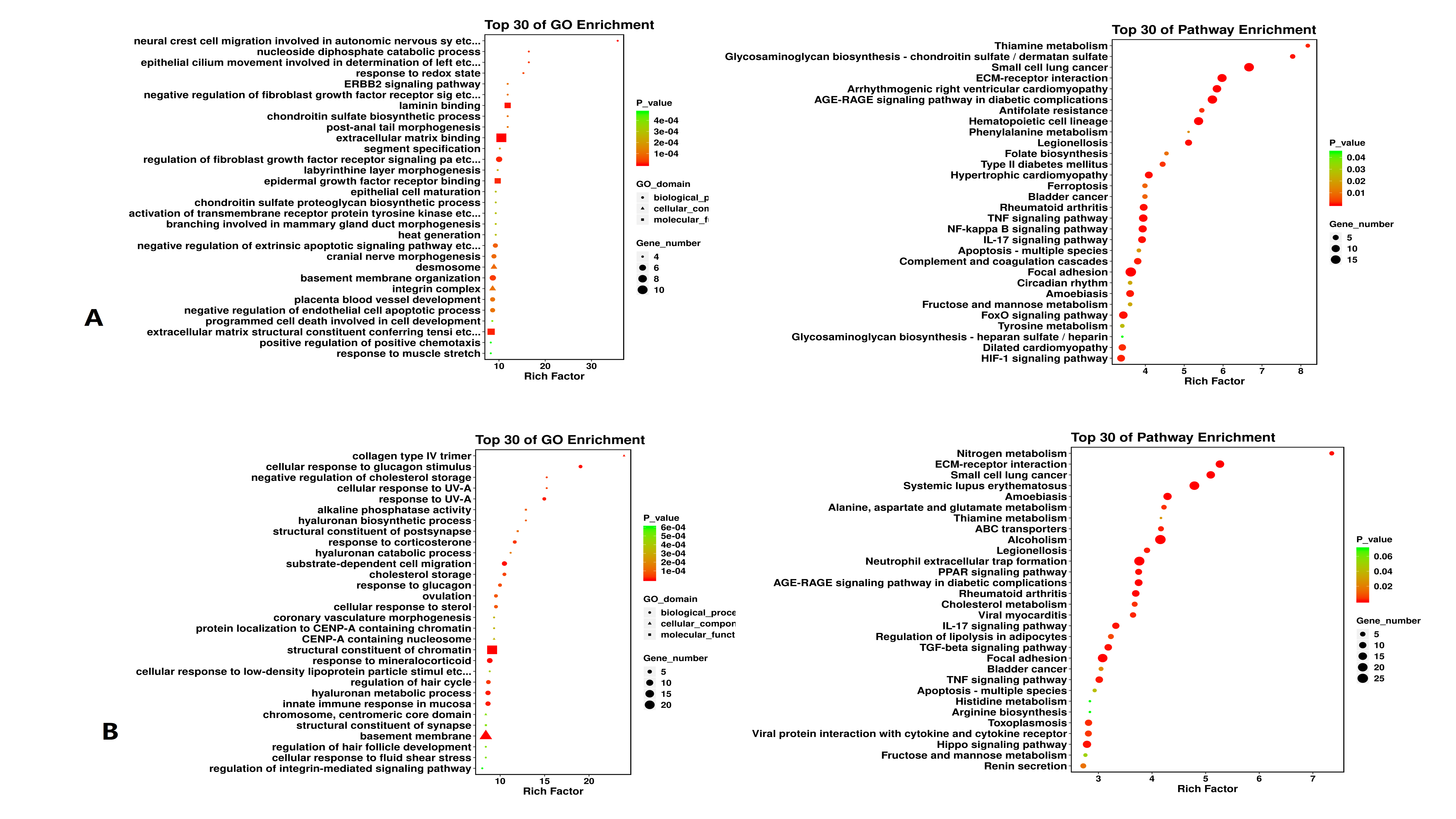

Supplement: Supplementary file 7 — Supplementary Material 7 [file 41598_2025_5435_MOESM7_ESM.png]

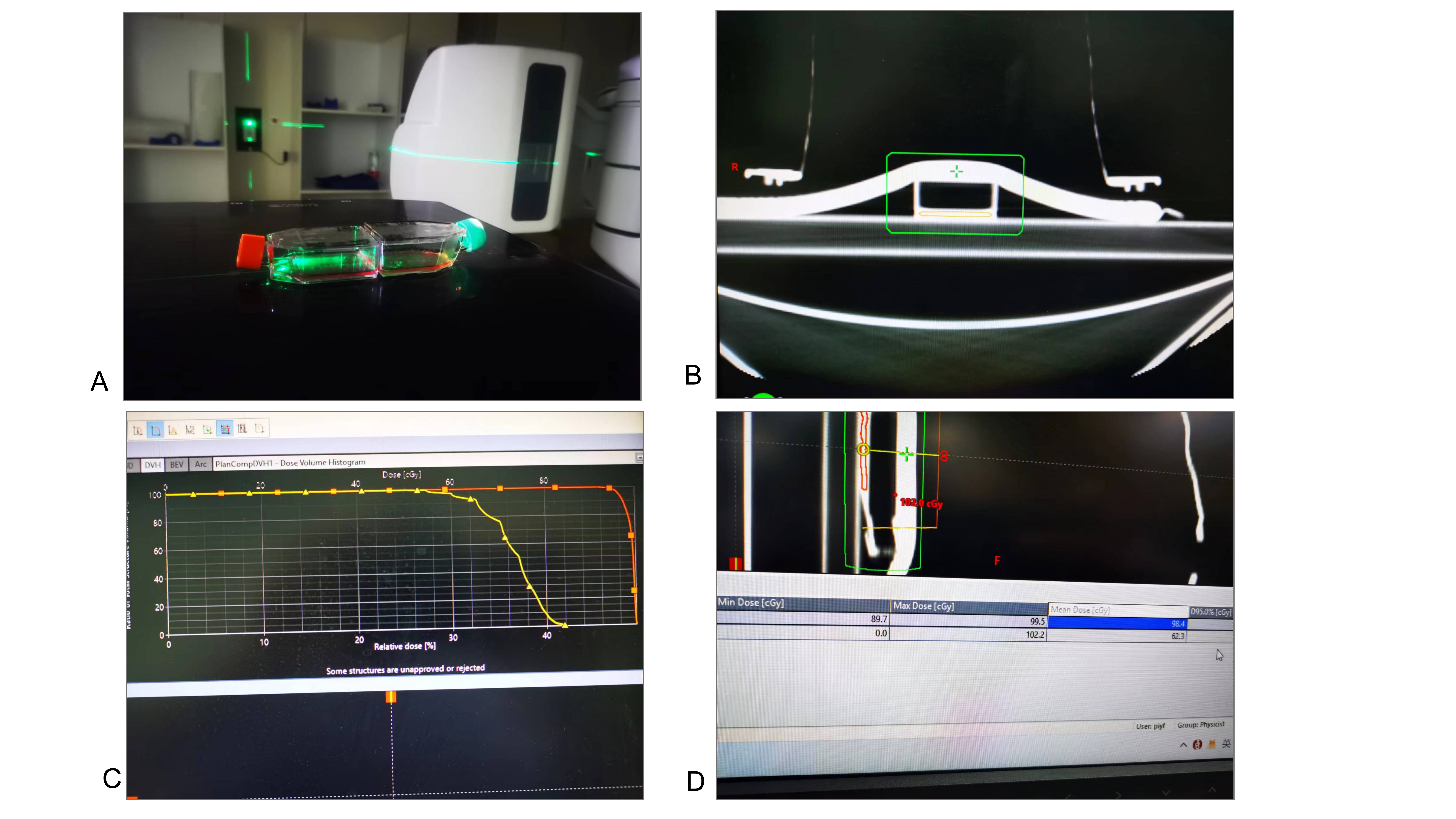

Supplement: Supplementary file 8 — Supplementary Material 8 [file 41598_2025_5435_MOESM8_ESM.png]

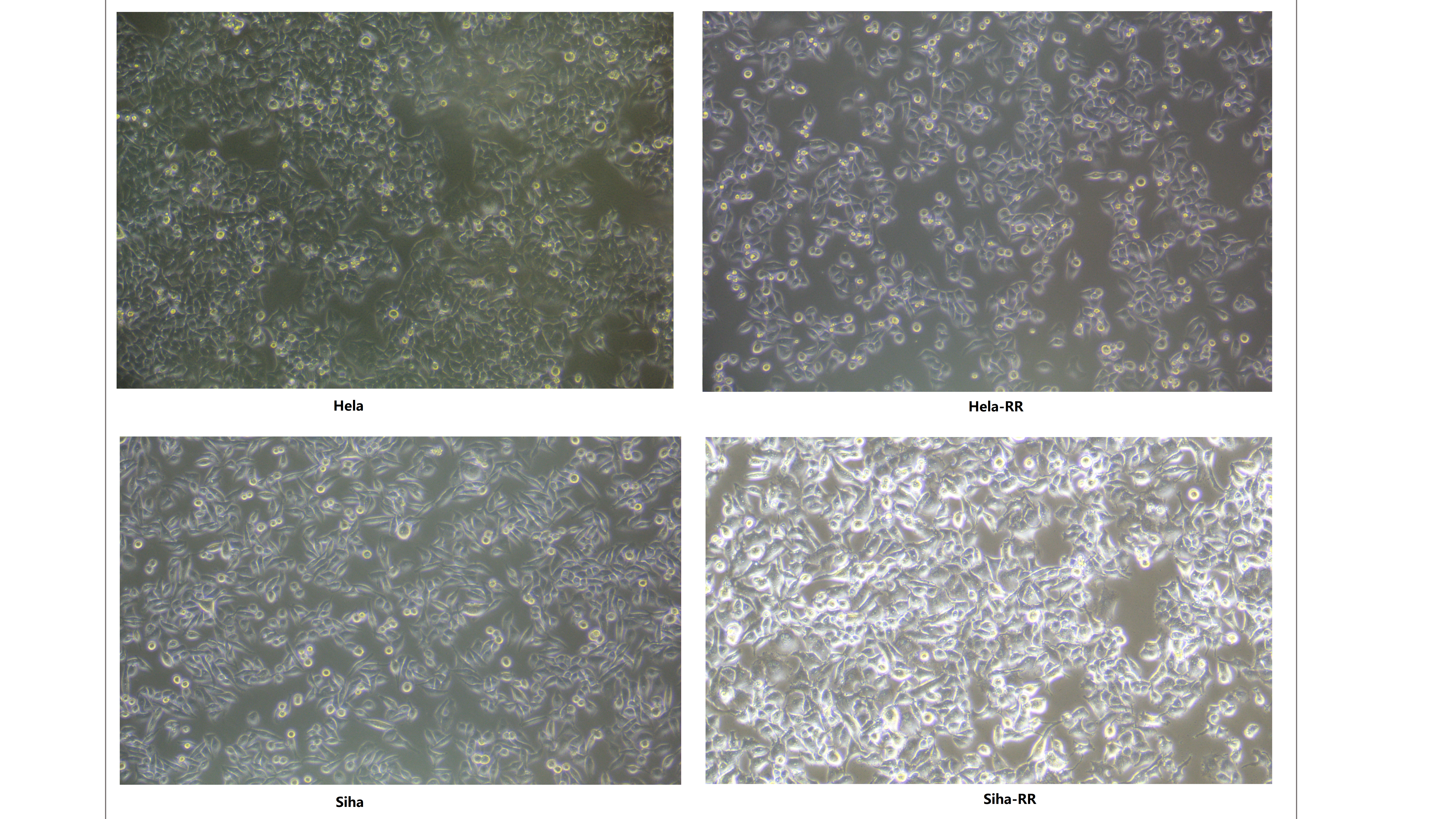

Supplement: Supplementary file 10 — Supplementary Material 10 [file 41598_2025_5435_MOESM10_ESM.png]

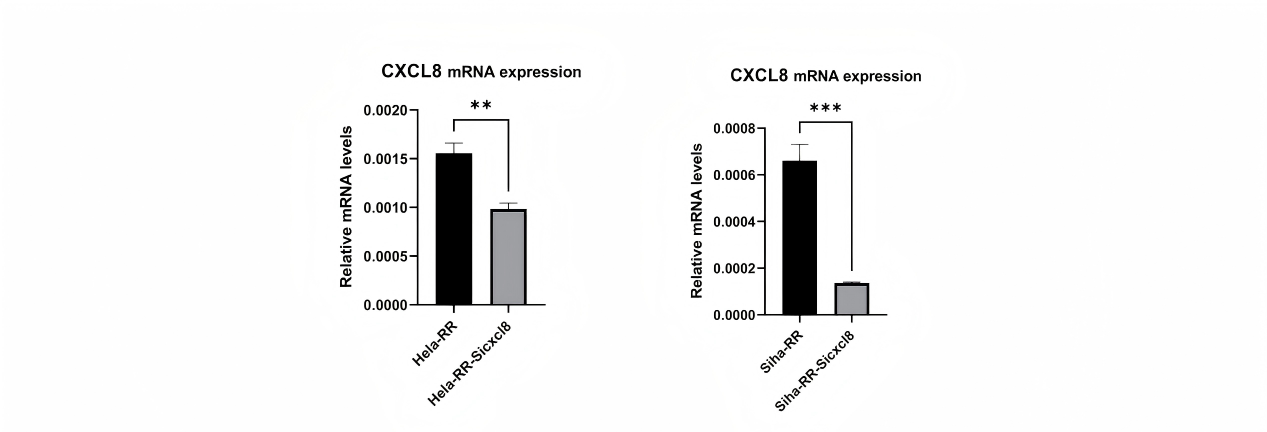

Supplement: Supplementary file 11 — Supplementary Material 11 [file 41598_2025_5435_MOESM11_ESM.png]

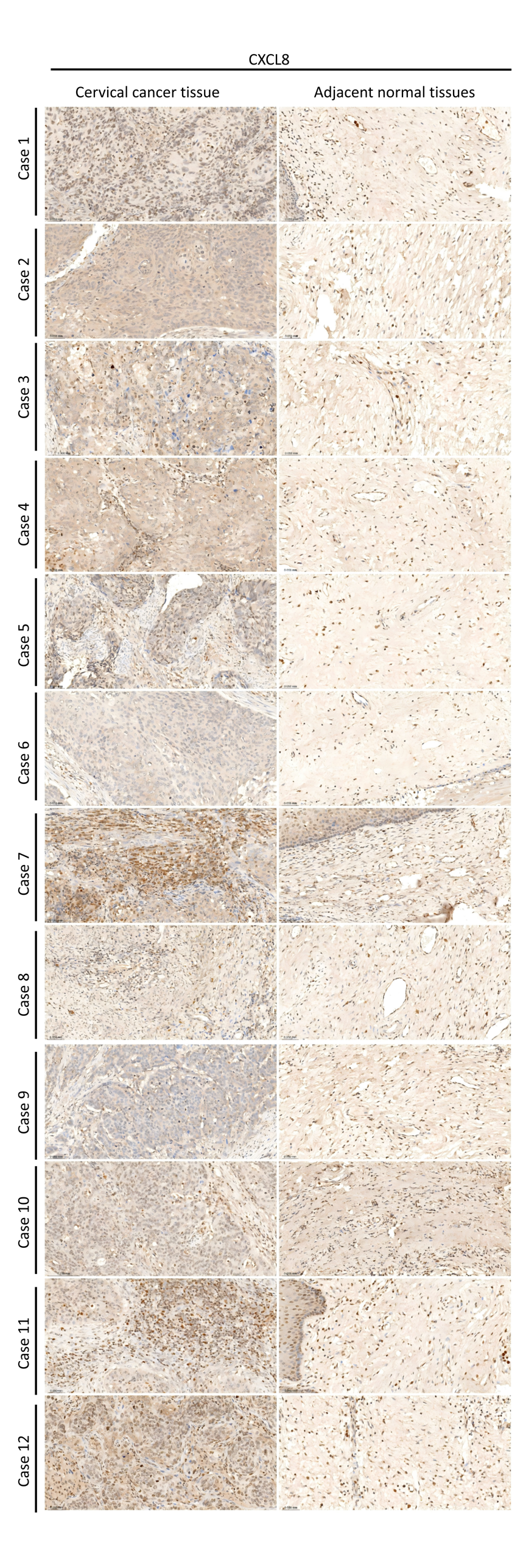

Supplement: Supplementary file 12 — Supplementary Material 12 [file 41598_2025_5435_MOESM12_ESM.png]
